# Behavioral Response to Catecholamine Depletion in Individuals With Schizophrenia and Healthy Volunteers

**DOI:** 10.1093/schizbullopen/sgad023

**Published:** 2023-08-24

**Authors:** Samir Suker, Yoan Mihov, Andreas Wolf, Stefanie V Mueller, Gregor Hasler

**Affiliations:** Psychiatric University Hospital, University of Bern, Bern, Switzerland; Unit of Psychiatry Research, University of Fribourg, Fribourg, Switzerland; Psychiatric University Hospital, University of Bern, Bern, Switzerland; Psychiatric University Hospital, University of Bern, Bern, Switzerland; Unit of Psychiatry Research, University of Fribourg, Fribourg, Switzerland

## Abstract

**Background and Hypothesis:**

Dysfunction of the dopamine system is the leading neurobiological hypothesis of schizophrenia. In this study, we tested this hypothesis in the context of aberrance salience theory of delusions using catecholamine depletion. We hypothesized that acute dopamine depletion improves both positive symptoms and salience attribution in individuals with schizophrenia.

**Study Design:**

Catecholamine depletion was achieved by oral administration of alpha-methyl-para-tyrosine (AMPT) in 15 individuals with schizophrenia and 15 healthy volunteers. The study design consisted of a randomized, double-blind, placebo-controlled crossover, single-site experimental trial. The main outcome measures were the Scale for the Assessment of Positive Symptoms and the Salience Attribution Test.

**Study Results:**

Catecholamine depletion transiently reduced specific psychotic symptoms in symptomatic individuals with schizophrenia, namely delusions and positive formal thought disorder (interaction treatment-by-timepoint, *P* = .013 and *P* = .010, respectively). We also found trends for catecholamine depletion to increase relevant bias and adaptive salience in participants with schizophrenia while decreasing them in healthy controls (interaction group-by-treatment, *P* = .060 and *P* = .089, respectively). Exploratory analyses revealed that in participants with schizophrenia, higher relevant bias at 3 hours after the end of AMPT treatment corresponded to lower delusional symptoms (Spearman’s rho = −0.761, *P* = .001).

**Conclusions:**

This study suggests that the relationship between dopamine hyperactivity and delusional symptoms in schizophrenia is mediated by impaired attribution of salience to reward-predicting stimuli.

## Introduction

Dysfunction of the dopamine system is the leading neurobiological hypothesis of schizophrenia. Initially, this hypothesis was based on findings that amphetamine could cause psychotic symptoms in otherwise healthy people and that all antipsychotic substances work by blocking dopamine receptors. Recent imaging studies in people with schizophrenia and in people at risk have consistently found that the major dopaminergic abnormality in schizophrenia is increased presynaptic activity in the striatum.^1^ While the dopamine dysfunction may be complex and heterogeneous in schizophrenia, elevated dopamine synthesis and release capacities have appeared to be core features of the disorder.^2–4^ Ellinwood’s hypothesis that suspiciousness and paranoia are the most “‘dopamine dependent’” dimension of psychosis have been replicated by more recent research.^5^ Furthermore, dopamine dysfunction has been linked to altered cortical function during cognitive tasks.^6^

Dopamine depletion using alpha-methyl-para-tyrosine (AMPT) is an instructive paradigm to directly investigate the relationship between increased presynaptic dopamine activity and symptoms in schizophrenia.^5,7–12^ AMPT competitively inhibits tyrosine hydroxylase, the rate-limiting enzyme in catecholamine synthesis, and is, therefore, able to decrease catecholaminergic neurotransmission by depleting central dopamine and norepinephrine stores.^13–16^

In an early study, AMPT was effective in reducing psychotic symptoms in 6 patients with schizophrenia, after their treatment with a typical antipsychotic substance was reduced until a clear exacerbation of symptoms occurred.^10^ Another early study found no change in psychotic symptoms when adding AMPT to a previously stable and theoretically adequate typical antipsychotic substance in 8 patients with treatment-resistant schizophrenia.^11^ In combination with molecular imaging, administration of AMPT resulted in a larger increase in dopamine-2 (D2) receptor availability in 18 untreated individuals with schizophrenia (19%) compared to healthy volunteers (9%), independent of previous treatments with antipsychotic substances.^5^ In addition, higher AMPT-induced increase in D2 receptor availability corresponded to a higher reduction of positive symptoms (*r* = 0.6). These findings provided direct evidence of increased stimulation of D2 receptors by dopamine in schizophrenia, consistent with increased capacity to synthesize and release dopamine, which may already be present in people with Ultra-High Risk for psychosis.^12^

More recent studies emphasized AMPT-induced dysphoric and depressive symptoms in schizophrenia,^8^ and their association with dopamine-related attribution of salience to reward-predicting stimuli.^9^ These AMPT effects may be of interest to understand the affective and cognitive side effects of continuous antipsychotic treatment that converts dopamine neurons into a condition of depolarization block, in which dopamine synthesis and release decline.^17^ Because of AMPT’s dysphoric side effect and its potential to induce akathisia and extra-pyramidal symptoms, it cannot be used as an antipsychotic medication. Particularly, restlessness and akathisia are more severe than observed with current antipsychotics.

The neuropsychological underpinnings of AMPT-induced increases and decreases of symptoms in schizophrenia have not yet been fully elucidated. Striatal dopamine synthesis-related aberrant salience is another putative neuropsychological substrate in schizophrenia,^18,19^ which may explain how a complex array of imaging findings and cognitive impairments converge neurochemically to cause psychosis through aberrant salience. So far, this hypothesis has not yet been tested using AMPT.

Given the discrepancies in the literature on AMPT depletion in schizophrenia and the lack of data on AMPT’s influence on core biomarkers of psychosis, we investigated the role of dopamine in different symptom domains of schizophrenia using symptom ratings and neuropsychological tasks in patients and healthy controls during AMPT-induced catecholamine depletion in comparison to a placebo control condition. In particular, we were interested in the relationship between dopamine neurotransmission and delusion-related salience alterations as assessed with the salience attribution task (SAT),^20^ working memory as assessed using the N-back-task, and cognitive and attentional flexibility as assessed using the intra–extra-dimensional set shift-task (IDED).^21^ To assess behavioral components of negative symptoms, we assessed physical effort using a handgrip-force paradigm.^22^

We were particularly interested in the SAT since there is increasing evidence that attribution of abnormally heightened salience to daily-life stimuli is associated with positive symptoms in psychosis.^23^ The salience attribution test (SAT)^20^ is a modified version of the monetary incentive delay task,^24^ where study participants are confronted with a series of stimuli presented in 2 dimensions such as color (red or blue) and shape (building or animal). Only one of the dimensions is relevant to reward. Salience attribution can be experimentally measured by reaction time (“bias)” and by self-report on a visual analog scale (“salience”). Attention to the reward-relevant dimension is called relevant bias and adaptive salience, whereas attention to the irrelevant dimension is referred to as irrelevant bias and aberrant salience. Clinical research using the SAT revealed that schizophrenia patients with delusion showed significantly reduced relevant bias and adaptive salience than healthy controls.^20^

## Methods

### Participants

Fifteen male persons with schizophrenia and 15 male healthy controls were included in this study. Participants with schizophrenia were recruited at the inpatient and outpatient departments of the Psychiatric University Hospital Bern, Switzerland, where the study was conducted. Healthy volunteers were recruited by advertisements in local newspapers and by announcements at the University of Bern. Controls were matched for age and duration of education. Before participants provided written informed consent, the study was fully explained to them. The protocol and the written informed consent were approved by the local ethics committee of Canton Bern, Switzerland, and were performed in accordance with the principles of the Declaration of Helsinki. During the screening visit, all participants underwent the Structural Clinical Interview for DSM-IV^25^ (SCID), a physical examination, a diagnostic interview with a psychiatrist, and filled out clinical questionnaires. Exclusion criteria for both groups included current Axis II psychiatric disorder, major medical or neurological illness, lifetime history of substance dependency, suicidal ideations within the last 4 weeks before and during study participation, and a history of suicide attempts. Clinical diagnoses of individuals with schizophrenia were made according to the DSM-IV criteria by a trained psychiatrist. All patients included met the criteria of the paranoid or disorganized subtypes of schizophrenia.

All healthy individuals were interviewed by a physician using the SCID. None of the healthy participants had a history of psychiatric disorders, any first-degree relatives with schizophrenia or schizoaffective disorder, any medical conditions affecting brain function, substance or alcohol abuse, none were using any medication at the time of testing.

Since our main hypothesis is differentially related to positive and negative symptoms of schizophrenia, we used the Scale for the Assessment of Positive Symptoms (SAPS)^26^ and the Scale for the Assessment of Negative Symptoms (SANS)^27^ at baseline and to evaluate the response to treatments. Given that previous studies reported mood and anxiety symptoms as response to AMPT in schizophrenia, we also applied the Montgomery-Asberg Depression Rating Scale (MADRS), which is a structured clinical interview,^28^ and the Beck Anxiety Inventory (BAI),^29^ which is a self-report measure. In addition, for assessment of executive function, we used the Mehrfachwahl–Wortschatztest (MWT-A) and the Digit-Symbol-Substitution subtest (DSST). Demographic and clinical characteristics are presented in table 1.

**Table 1.** Sociodemographic and Clinical Characteristics

Variable	Healthy Controls (*N* = 15)		Participants with Schizophrenia (*N* = 15)		Comparison	
	Range	Mean (*SD*)	Range	Mean (*SD*)	Test Statistic	*P*
Age	20–51	30.867 (7.744)	19–60	30.4 (10.802)	*t* = 0.136	.893
Weight	60–100	75.929 (9.724)	65–107	83.64 (14.602)	*t* = −1.684	.105
Years of education	9–18	11.8 (3.448)	9–15	10.75 (2.22)	*t* = 0.957	.348
MWT-B	24–35	30.73 (3.03)	23–34	29.20 (2.76)	*t* = 1.449	.159
DSST	46–60	52.6 (4.66)	31–62	46.53 (9.72)	*t* = 2.18	.041
CPZ Equiv.			0–1203	300.07 (303.24)	—	—
Smokers	8 out of 15		13 out of 15		OR = 5.353	.109
Cigarettes/d	0.25–20	12.531 (8.634)	2–30	15.538 (7.677)	*t* = −0.808	.433
Alcohol	13 out of 15		7 out of 15		OR = 0.145	.05
Glasses/w	0.5–6	3.346 (1.772)	0.25–6.5	1.536 (2.21)	*t* = 1.868	.091
Cannabis	2 out of 15		6 out of 15		OR = 4.123	.215
Cannabis/w	0.125–0.5	0.313 (0.265)	0.25–7.5	1.875 (2.792)	*t* = −1.353	.231

***Note:*** SD, refers to the standard deviation; Test statistic: *t*, refers to the *t*-value from Welch 2 sample *t*-test; OR, refers to the odds ratio and Fisher’s exact test for count data; *P*, refers to the *P*-value in 2-tailed tests; MWT-B, refers to the sum of correct responses in the verbal test Mehrfachwahl–Wortschatztest, version B; DSST, refers to the number of correct responses in the Digit Symbol Substitution subtest of Wechsler Adult Intelligence Scale; CPZ Equiv, corresponds to chlorpromazine equivalent dose; Cigarettes/ d, refers to cigarettes smoked per day in smokers; Alcohol, refers to the number of participants drinking alcohol; Glasses/w, refers to the alcohol consumption in number of glasses per week in participants drinking alcohol; Cannabis, refers to the number of participants consuming cannabis; Cannabis/w, refers to the number of occasions of cannabis consumption per week in participants consuming cannabis; Sample size = 30 (15 healthy controls and 15 participants with schizophrenia), except for weight, where *n* = 29 (14 healthy participants and 15 participants with schizophrenia); years of education, where *n* = 27 (15 healthy controls and 12 participants with schizophrenia); chlorpromazine equivalent dose, where *n* = 14 (missing data for one participant with schizophrenia).

### Study Design and Dopamine Challenge

**Fig. 1.**
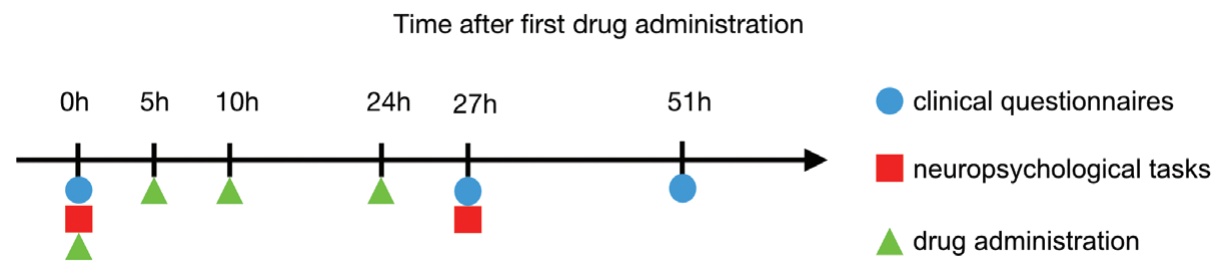
An overview of the study design.

The study included a screening visit and 2 experimental sessions (figure 1). In a randomized, double-blind, placebo-controlled, crossover design, study participants underwent 2 identical sessions separated by at least one week to avoid any crossover effects. In the treatment condition, participants received orally alpha-methyl-para-tyrosine (AMPT) in a body-weight adjusted dose over 24 hours: On day 1 at 9 AM, at 2 PM, and at 7 PM and on day 2 at 9 AM. In the sham condition, the participants received 25mg of diphenhydramine at the first and placebo at the remaining 3-time points. Diphenhydramine was administered in the sham condition to increase blinding between the 2 sessions because compared to AMPT it induces similar sedation, but no other symptoms.^30,31^ To reduce the risk of adverse reactions, we used a body weight–adjusted AMPT dose of 40mg/kg of body weight, to a maximum of 4g, over 24 hours. These doses were similar to those used by Hasler et al^16^ To reduce the risk of crystalluria during AMPT administration, participants were instructed to drink at least 2l of water daily, and underwent urinalysis prior to AMPT administration.

### Neuropsychological Tasks

Twenty-seven hours after administration of AMPT or placebo participants completed a series of neuropsychological tasks comprising the salience attribution test (SAT), the N-back task, the intradimensional/extradimensional set-shifting task (IDED), and the handgrip-force task (figure 1). All tasks were presented on a laptop except for IDED, which was carried out on a tablet. Detailed task descriptions can be found in the Supplementary Information.

### Hypothesis and Subhypotheses

The main hypothesis was that in participants with schizophrenia AMPT-induced catecholamine depletion reduces positive symptoms at the cost of exacerbating dysphoric mood and—when probed with experimental tasks—executive, cognitive and motivation deficits. In healthy controls, without schizophrenia symptoms, AMPT have a similar but lower impact on executive function and motivation. From this main hypothesis, a series of subhypotheses regarding specific clinical measures and neuropsychological tasks can be derived, as follows.

With respect to SAPS and SANS, we expected to find positive and negative symptoms in participants with schizophrenia, but not in healthy controls. From the main hypothesis, the following subhypothesis can be derived: In the schizophrenia group AMPT improves positive, but not negative, symptoms and this effect subsides over the course of testing. This hypothesis translates to a treatment-by-timepoint interaction in an analysis of variance with the factors treatment (AMPT vs. placebo) and timepoint (baseline vs. 27 hours post vs. 51 hours post).

We expected that participants with schizophrenia would report higher symptoms of depression and anxiety, as measured with MADRS and BAI, respectively. In an ANOVA with the factors group (schizophrenia vs. healthy controls), timepoint (baseline vs. 27 hours post vs. 51 hours post), and treatment (AMPT vs. placebo) the expected between-group difference would manifest as a main effect of group. From the main hypothesis, the following subhypotheses can be derived: AMPT further exacerbates MADRS and BAI scores temporarily (interaction treatment-by-timepoint) and has a higher impact in the group with schizophrenia (interaction treatment-by-timepoint-by-group).

In the SAT, we expected persons with schizophrenia to exhibit more irrelevant bias and aberrant salience than healthy controls, as reflected by a main effect of group in ANOVA with the factors group (schizophrenia vs. healthy controls) and treatment (AMPT vs. placebo). From the main hypothesis, the following sub-hypothesis can be derived: AMPT improves both irrelevant bias and aberrant salience in participants with schizophrenia but not in healthy controls (interaction treatment-by-group on the parameters irrelevant bias and aberrant salience). In addition, we ran exploratory analyses on adaptive salience and relevant bias.

In the handgrip-force task, we expected that all participants will choose the more difficult option more frequently as the probability of winning or the monetary reward increases, corresponding to a main effect of task condition in ANOVAs with the factors condition either winning probability (13%, 59%, and 87%), or amount (2, 5, or 10) as dependent variables and the factors group (schizophrenia vs. healthy controls) and treatment (AMPT vs. placebo). We expected that participants with schizophrenia will choose the harder option less frequently overall (main effect of group). From the main hypothesis, the following subhypotheses can be derived: AMPT decreases the tendency to choose the harder options overall (main effect of treatment) and this effect is stronger in the group with schizophrenia (interaction treatment-by-group).

In the n-back task, the main outcome measure we analyzed was the number of correct responses (hits) in an ANOVA with the factors task condition (0-back vs. 1-back vs. 2-back vs. 3-back), group (schizophrenia vs. healthy controls), and treatment (AMPT vs. placebo). In addition, we ran exploratory analyses with the same factors and the number of false alarms, false negatives, and true negatives as dependent variables. We expected that the number of hits decreases with increasing cognitive load in both groups (main effect of task condition). From the main hypothesis, the following subhypotheses can be derived: AMPT treatment increases the number of hits (main effect of treatment), and this effect is stronger in the group with schizophrenia (interaction treatment-by-group).

In the IDED we expected the healthy group to perform better, reflected by a lower number of trials needed to reach criterion (main effect of group) in an ANOVA with the factors stage (to account for different stages of the task), group (participants with schizophrenia vs. healthy controls), and treatment (AMPT vs. placebo). From the main hypothesis the following hypotheses can be derived: AMPT impairs functioning, thus, increasing the number of trials to reach criterion (main effect of treatment) and this effect is stronger in the group with schizophrenia (interaction treatment-by-group).

### Statistical Analysis

Statistical analysis was carried out with R, version 4.1.3. We used the R-package “ez,” version 4.4.0, to calculate analyses of variance (ANOVAs). ANOVAs were calculated with type III sums of squares. For calculation of effect sizes in “ez,” the factor “Group” was declared as an observed, non-manipulated factor, whereas task condition and pharmacological treatment represented experimentally manipulated factors. Figures were plotted with the R-packages “lattice,” version 0.20.45, and “gridExtra.”^32,33^ We reported exact test statistics (such as F-values), exact *P*-values for all effects and interactions and supplemented these results with generalized effect size measures (see supplementary information).

We did not correct for multiple comparisons. A stringent correction for multiple comparisons for 4 clinical rating scales and 4 neuropsychological tasks (even for a single outcome parameter in each task) would strongly reduce the statistical power and incur high levels of beta risk or, alternatively, require very large sample sizes that would be hard to recruit and test in a within-subject pharmacological probe design. Multiple testing results in an increase in alpha risk (false positives) and, thus, our findings have to be interpreted with caution.

## Results

### Study Sample

A total of 32 participants provided written informed consent. Two of them, both with schizophrenia, dropped out of the study due to a lack of motivation. A summary of sociodemographic and clinical variables is presented in table 1. Healthy controls and participants with schizophrenia did not differ significantly in their age, weight, and years of education (table 1). Both groups did not differ in their performance in the Mehrfachwahl-Wortschatz-Intelligenztest-B (MWT-B), but the healthy controls scored better in the DSST (*P* = .041, 2-tailed, table 1). Both groups did not differ significantly in the number of smokers or the number of cigarettes smoked daily by smokers (table 1). Moreover, both groups did not differ in the number of participants consuming cannabis or the number of occasions of cannabis consumption per week in participants consuming cannabis in each group (table 1). We found nonsignificant trends towards a higher number of participants consuming alcohol in healthy controls, as compared to participants with schizophrenia (*P* = .05, 2-tailed, table 1), and a higher alcohol consumption in those consuming alcohol in controls than in participants with schizophrenia (*P* = .09, 2-tailed, table 1). Data assessments for this study took place from November 2014 to June 2016.

### Medication

Thirteen out of fifteen participants with schizophrenia received medication during their participation in the experiment. Five of those who received more than one medication. Four participants received olanzapine, 4 quetiapine, 3 risperidone, 2 clozapine, 1 amisulpride, 1 pipamperone, 1 paliperidone, 2 valproate, 1 fluvoxamine, 1 lisinoprile, 1 propranolol, and 1 pantoprazole.

### Scale for the Assessment of Positive Symptoms/Scale for the Assessment of Negative Symptoms

Whereas participants with schizophrenia displayed various levels of positive and negative symptoms throughout the experiment, healthy controls showed none, with the exception of a single healthy control that displayed transient attention problems at baseline (supplementary figures 3 and 7). We did not analyze the scores of healthy participants and participants with schizophrenia in a joint ANOVA due to the lack of variance in healthy participants.

**Fig. 2.**
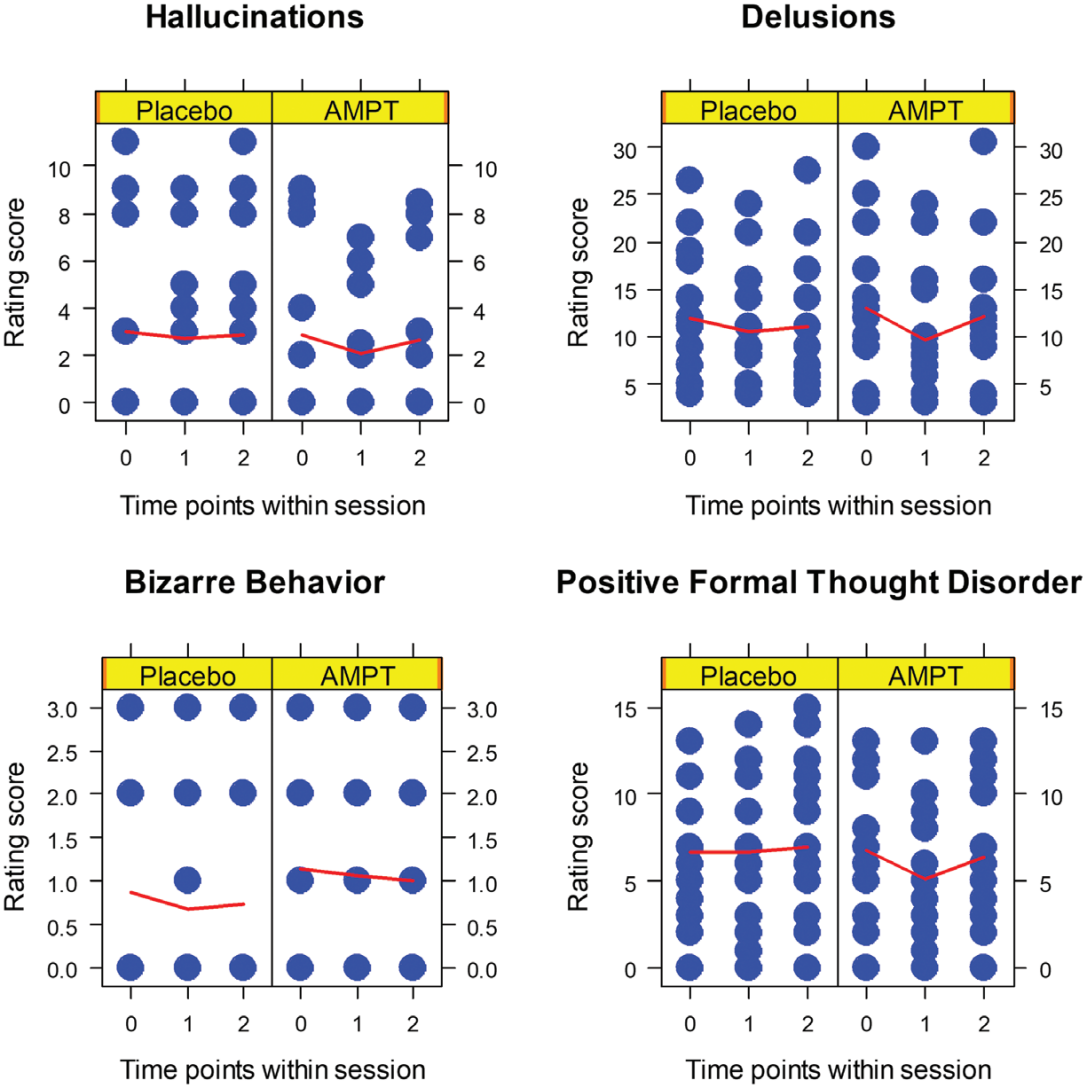
SAPS In Participants With Schizophrenia. *Blue dots* represent rating scores of individual participants; *red lines* connect group averages per timepoint; *time points* as follows: 0 = baseline, 1 = 27 hours after the begin of alpha-methyl-para-tyrosine (AMPT)/placebo administration, 2 = 51 hours after the begin of AMPT/placebo administration; *sample size*, *n* = 15 participants with schizophrenia.

In participants with schizophrenia AMPT temporarily reduced positive formal thought disorder and delusions (supplementary table 1). AMPT treatment did not significantly alter other positive symptoms, as measured with the SAPS (supplementary table 1). SAPS scores for “Delusions” and “Positive Formal Thought disorders” dropped from baseline to 3 hours and increased again at 27 hours after the last treatment with AMPT, but not placebo (figure 2, supplementary figures 1–3). Correspondingly, we found a significant main effect of timepoint and a significant interaction treatment-by-timepoint for these 2 scales (supplementary table 1). We did not find other significant main effects and interactions in SAPS (supplementary table 1). Overall, our treatment subhypotheses were corroborated for some, but not all, positive symptom measures. In participants with schizophrenia, in line with our sub-hypothesis, we found no significant main effects and interactions for SANS (supplementary table 2, supplementary figures 4–7).

Healthy participants, as expected, displayed no positive or negative symptoms of schizophrenia and this did not change over the three timepoints for either placebo or AMPT (supplementary figures 3 and 7).

### MADRS and BAI

As expected, participants with schizophrenia reported higher MADRS scores than controls, as reflected by a significant main effect of group (supplementary tables 3, figure 8). Increased MADRS scores were recorded in the session with AMPT treatment, corresponding to a significant effect of treatment (supplementary table 3, figure 8). MADRS scores changed over the three timepoints around each treatment (a significant main effect of timepoint, supplementary table 3, figure 8). A nonsignificant trend group-by-treatment suggested that the effect of AMPT was stronger in participants with schizophrenia, corroborating our sub-hypothesis (*P* = .09, supplementary table 3). In disagreement with our sub-hypothesis, we did not find a significant interaction group-by-timepoint-by-treatment and, hence, no evidence that the difference in AMPT treatment between participants with schizophrenia and healthy controls significantly varied over timepoints (supplementary table 3).

As expected, participants with schizophrenia also reported higher BAI scores than controls, reflected by a significant main effect of group (supplementary tables 4, supplementary figure 8). We found no significant main effect of treatment on BAI scores (supplementary table 4, figure 8). BAI scores changed over the 3 timepoints around each treatment (a significant main effect of timepoint, supplementary table 4). A nonsignificant trend suggested that the effect of AMPT differed between participants with schizophrenia and controls (interaction group-by-treatment, *P* = .07); however, this might be due to outlier BAI values at baseline in the schizophrenia group (supplementary table 4, figure 8). We found no other significant interactions (supplementary table 4). Overall, our subhypotheses were only partly corroborated by BAI and MADRS findings.

### SAT

**Fig. 3.**
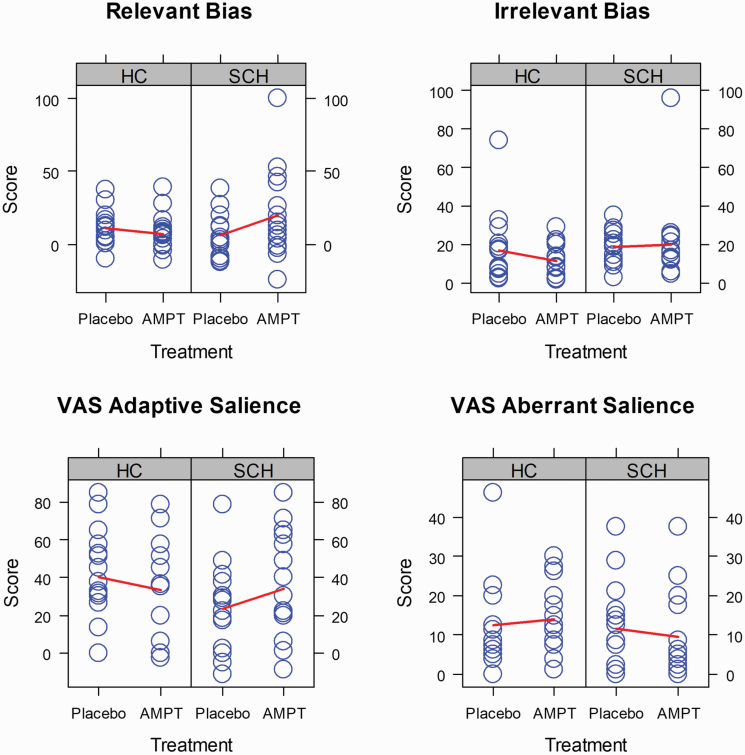
Performance in the salience attribution task. *Blue circles* represent individual participants; *red lines*, connect group averages under placebo and alpha-methyl-para-tyrosine treatment; *sample size*, *n* = 30 participants (15 healthy controls and 15 participants with schizophrenia).

Other than expected, we found no significant main effects and interactions in the SAT (figure 3, supplementary tables 5–8). However, we found a trend for significance for 2 group-by-treatment interactions that we consider consistent, relevant and, hence, noteworthy. These interactions suggest that AMPT decreased relevant bias (implicit measures) and adaptive salience (explicit measures) in controls but increased them in participants with schizophrenia (figure 3). The corresponding *P*-values were *P* = .06 for relevant bias and *P* = .09 for adaptive salience (supplementary tables 5 and 6).

An exploratory analysis revealed that in participants with schizophrenia (*n* = 15) 3 hours after the last AMPT administration relevant bias in the SAT was negatively correlated with the Delusions score in SAPS (Spearman’s rho = −0.76, *P*-value < .001, supplementary figure 9). In the same group (*n* = 15), both measures did not correlate significantly 3 hours after placebo administration or at baseline before placebo administration (supplementary figure 10).

### Handgrip-Force Task

In the force task, both monetary rewards and the probability of winning influenced behavior as expected (supplementary figures 11–14). For increasing monetary rewards participants from both groups chose more often the hard option (supplementary figures 11 and 12). Likewise, for increasing probability of winning, both groups chose more often the hard option (supplementary figures 13 and 14). Correspondingly, we found a significant main effect of monetary reward and probability of winning (supplementary tables 9 and 10). As expected, controls chose the hard option more frequently than participants with schizophrenia regardless of probability of winning and payoff, reflected by a significant main effect of group (supplementary figures 11, 14). In disagreement with our subhypotheses, we found no effect of AMPT treatment and no interactions between group, treatment, and monetary reward or winning probability (supplementary tables 9 and 10).

### N-back Task

One participant with schizophrenia was excluded after completing only one session and scoring 0 correct responses for the conditions “2-back” and “3-back,” resulting in *n* = 15 healthy controls and *n* = 14 participants with schizophrenia.

**Fig. 4.**
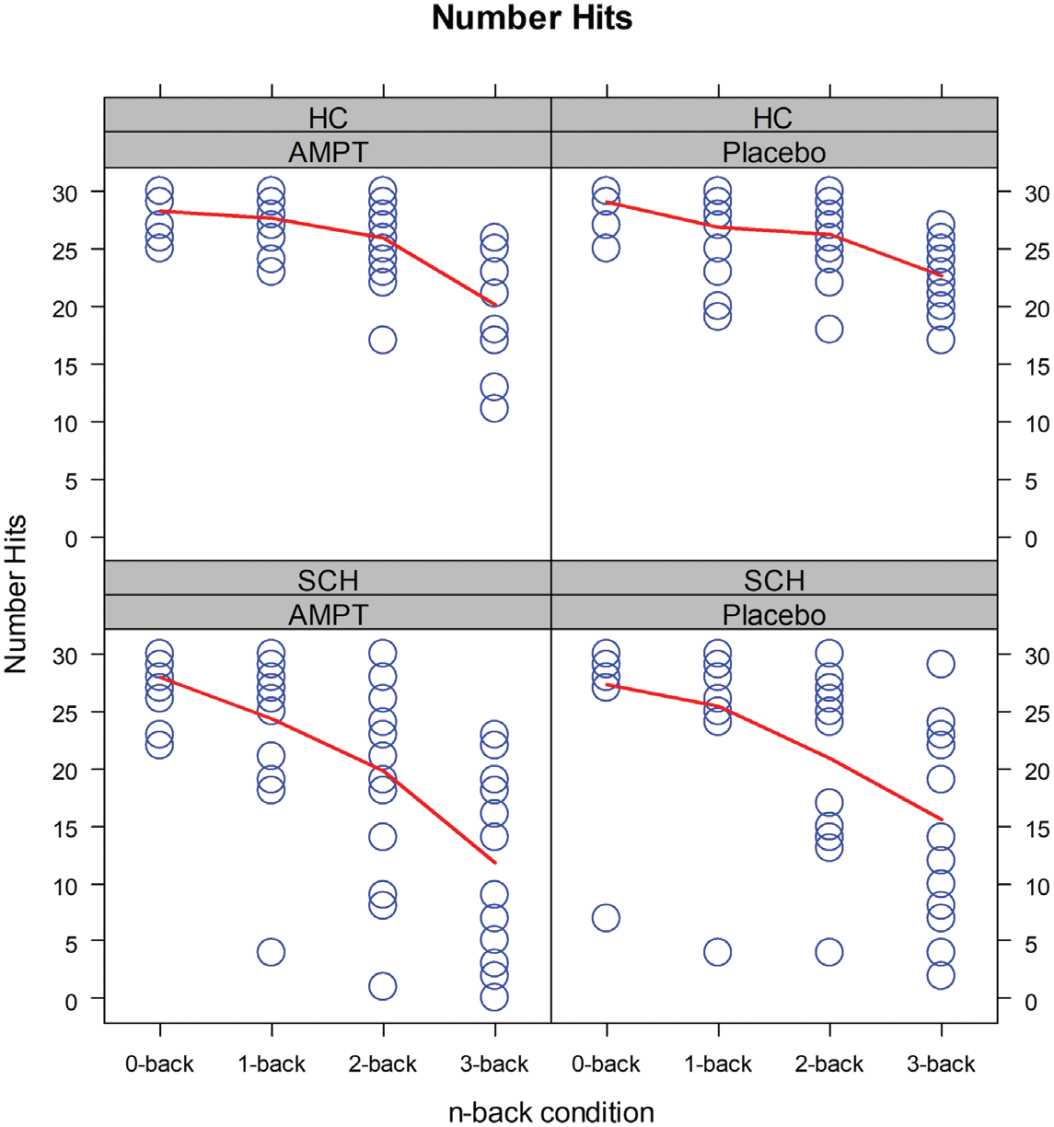
Performance in the N-back Task. Number of Hits. *Blue circles* represent individual participants; *red lines* connect group averages across conditions; *sample size*, *n* = 29 participants (15 healthy controls and 14 participants with schizophrenia).

When *n*-back performance was measured with the number of hits performance deteriorated significantly as cognitive load increased from 0-back to 3-back, corresponding to the expected significant main effect of condition (figure 4, supplementary table 11). As expected, overall, healthy controls performed better than participants with schizophrenia, reflected by a main effect of group (figure 4, supplementary table 11). The increasing cognitive load affected hits more strongly in participants with schizophrenia than in controls (figure 4). Correspondingly, we found a significant group-by-condition interaction (supplementary table 11). In disagreement with our sub-hypothesis, we did not find an overall effect of AMPT on performance and no interaction group-by-treatment, but a significant treatment-by-condition interaction indicating that AMPT reduced the number of hits for higher cognitive loads in both groups in a similar manner (figure 4, supplementary table 11). We found no evidence for a complex treatment-by-condition-by-group interaction (supplementary table 11).

The results we found for hits were mirrored by the results for false negatives (supplementary table 14, figure 17). We found a significant increase in false negatives for increasing cognitive demand. Controls performed overall better than participants with schizophrenia, and this was more strongly pronounced for higher cognitive demands (supplementary table 14, figure 17).

With respect to other performance measures in the *n*-back task, we found that increasing cognitive load led to a decrease in the number of true negatives and an increase in false alarms, as indicated by a significant effect of condition (supplementary tables 12/13, figures 15 and 16). For these performance measures, we found no further significant effects or interactions (supplementary tables 12 and 13).

### Intra–Extra-Dimensional Set Shift-Task

All participants successfully completed the first 7 stages, such that we only observed attrition in stages 8 and 9 (extra-dimensional shift and extra-dimensional shift reversal, respectively, supplementary tables 15/16). At stage 8, 5 healthy controls and 6 participants with schizophrenia failed to reach completion criteria (supplementary table 15). At stage 9, 2 healthy controls failed to reach completion criteria (supplementary table 15).

Due to failures to complete stages and missing data, we calculated an ANOVA on the data from the first 7 stages (no missing data, *n* = 30, 15 participants per group, supplementary table 17). In this analysis, the dependent variable was the number of trials, within-group factors were stage (1–7) and treatment (Placebo vs. AMPT), and the between-group factor was participant group (healthy controls vs. patients). We found a significant main effect of stage reflecting varying difficulty levels between stages (supplementary table 18, figure 18). We found a nonsignificant trend for the expected main effect of group, reflecting a tendency towards more errors in participants with schizophrenia (altogether 1461 trials in healthy controls and 1605 trials in participants with schizophrenia, *P* = .09, supplementary table 18). In disagreement with our subhypotheses, we found no significant effect of AMPT treatment and no significant interactions between stage, group, and treatment (supplementary table 18, figure 22).

### Association Between Symptoms and Cognitive Function

To assess the relation between negative and positive symptoms with cognitive function, we performed a comprehensive series of analyses (see supplementary tables 18–20). In summary, we found no significant relation between the magnitude of positive or negative schizophrenia symptoms and cognitive function, as measured with the MWT, DSST, and the n-back task.

## Discussion

In this study, we evaluated the effect of catecholamine depletion on various symptom dimensions in schizophrenia using the SPAS for positive symptoms, the SANS for negative symptoms, the MADRS for depressive symptoms, and the BAI for anxiety symptoms. In addition, we evaluated schizophrenia-related symptoms in behavioral experiments, namely, the Handgrip-Force Task for negative symptoms, and the N-back Task and the IDED for cognitive deficits. We demonstrated that catecholamine depletion using AMPT reduced specific psychotic symptoms in symptomatic patients with schizophrenia, namely delusions and positive formal thought disorder as assessed using the SAPS. We also found trends for AMPT to increase relevant bias and adaptive salience on the SAT, which have been related to positive psychotic symptoms. In addition, this study showed that AMPT did not change other psychotic symptoms and performance on the handgrip-force task, N-back task, IDED. Finally, there was a trend for AMPT-induced mood and anxiety symptoms being more pronounced in participants with schizophrenia than in controls.

Our findings are in line with Ellinwood’s hypothesis that suspiciousness/paranoia is the most “‘dopamine dependent’” dimension of psychosis^34^ and with more recent research that supports his hypothesis. Studies using amphetamine challenge in combination with PET imaging demonstrated significant correlations between positive psychotic symptoms and exaggerated dopamine transmission at D2 receptors in schizophrenia.^35^ A previous study using AMPT and PET imaging found that among positive symptoms, only severity of suspiciousness was associated at trend level with AMPT effect on D2 receptor availability.^5^ Another catecholamine depletion study also found that AMPT reduced psychotic symptoms, mainly positive symptoms, and general psychopathology.^8^ In people with Ultra-High Risk for psychosis, a SPECT study revealed that positive symptoms responded to AMPT and that higher synaptic dopamine concentration predicted larger reduction of positive symptoms following depletion.^12^ Our analysis of AMPT effects on depressive and psychotic symptom dimensions shows a relatively specific relationship between delusions and positive formal thought disorder and dopamine function. In contrast, in people with remitted major depression, AMPT-induced mostly depressive and anhedonic symptoms,^16^ related to performance deficits on a reward processing task.^36^

There is preliminary evidence that salience attribution is related to dopamine dysfunction in schizophrenia. An fMRI study in people at ultra-high risk for psychosis (UHR) and healthy controls showed that UHR was associated with enhanced aberrant salience and that the degree of aberrant salience correlated positively with neuronal activation in the ventral striatum across all study participants. In addition, in UHR, this activation correlated with the degree of delusional thought content.^37^ A longitudinal study using the SAT found higher irrelevant bias, higher aberrant salience and lower adaptive salience at baseline and lower relevant bias at follow-up in UHR compared to healthy controls.^38^ In addition, this study found that reduced activation of the ventral striatum during active reward prediction, suggesting an association between dopaminergic dysfunction and attribution of abnormally heightened salience to daily-life stimuli. In line, a study that combined SAT, operant learning task fMRI, and positron emission tomography in healthy volunteers revealed that reward prediction-error signal in the ventral striatum was negatively correlated with aberrant salience score, which was positively correlated with the striatal dopamine level.^19^ Although there is some discrepancy in the literature on salience attribution in schizophrenia, possibly due to heterogenic sample characteristics, the SAT has proved to be the best paradigm to study the relationship between delusion and dopamine neurotransmission.

Our study added to the previous literature on SAT that the relationship between dopamine neurotransmission and salience attribution, in terms of relevant bias and adaptive salience, may differ between schizophrenia patients and healthy controls. One might speculate that depending on the actual level of dopamine neurotransmission, being related to the exposure to stress and reward, salience attribution differs between patients and controls. These findings suggest more complex mechanisms of aberrant salience attribution underlying delusions in schizophrenia than previously assumed,^20^ which may explain some discrepant findings in previous studies. Interestingly, the AMPT-induced increase in relevant bias in the SAT correlated strongly with the AMPT-induced reductions in delusional symptoms, suggesting a relationship between normalization of dopamine function, improved focus on relevant stimuli, and anti-delusional effects found in our study.

Given that AMPT-induced depressive and ­anhedonic symptoms and deficits in reward processing in ­previous studies in people with depressive and eating disorders,^16,24,36,39–42^ we investigated the effect of AMPT on depressive and negative symptoms and experimentally measured anhedonia using the Handgrip-Force Task. As expected, we found increased levels of mood, anxiety, and negative symptoms and less willingness to perform in schizophrenia patients relative to controls. However, we did not find treatment-by-group interactions on any of these measures, suggesting that there may be no strong and direct relationship between dopamine neurotransmission, motivation-related reward functions, and negative symptoms in schizophrenia, which is consistent with the lack of efficacy of typical antipsychotics on negative symptoms.^43^

Schizophrenia is associated with premorbid generalized cognitive impairments that worsen throughout development of the illness.^44^ Among these impairments, working memory deficits and set-shifting deficits, ie, the reduced ability to move back and forth between different tasks or mental sets, have been associated with abnormal dopamine neurotransmission.^21,45^ As a result, we included the N-back Task to measure working memory and the IDED to measure set-shifting in this study. As expected, increasing cognitive load led to more working memory deficits in people with schizophrenia than in controls. However, we did not find a consistent relationship between catecholamine depletion and this difference. Regarding set shifting, we found a nonsignificant trend for more errors in participants with schizophrenia than in controls and no relationship to catecholamine depletion. Taken together, we did not find evidence for strong and direct relationships between cognitive deficits and dopamine neurotransmission in this study. Our findings are consistent with moderate and inconsistent effects of typical antipsychotics on cognitive functioning in schizophrenia^46^ and with the lack of consistent AMPT effects on working memory and attention in our previous study in people with remitted depression and healthy controls.^36^

Some limitations of our methods warrant comment. Patients with schizophrenia included in this study underwent an extensive study protocol with medication and multiple tasks. All of them were able to understand the whole protocol, give informed consent, and complete the whole protocol, ie, they were quite good-functioning patients at the moment of the trial, notwithstanding their suffering from symptoms. As a result, our study sample is not representative of all schizophrenia patients, especially not for those who do not agree with the healthcare professionals on explanatory model, diagnosis, and treatments. Our sample was rather small and findings regarding salience attribution should not be overinterpreted given that these fall beneath the threshold for statistical significance and there are multiple comparisons, which have not been corrected. In addition, AMPT’s effects of reducing the synthesis of norepinephrine as well as dopamine and the AMPT’s nonspecific induction of dysphoria limited the specificity of our results. Our cross-sectional design could not establish whether the antipsychotic response to AMPT in schizophrenia reflected an endophenotypic biomarker or a consequence of illness and its treatment. The correlation between the dopamine-related increase in relevant bias and reductions in delusional symptoms was not based on a specific hypothesis but the result of the general hypothesis, namely that AMPT-induced changes in the SAT are related to AMPT-induced reductions in positive symptoms. Moreover, we did not have a measure to determine the depth of catecholamine depletion. As a result, differences in depletion strength may have contributed to the variance of AMPT effects found in this study. Finally, we did not include neuroimaging measures that would have allowed for quantification of the AMPT effect in specific brain regions. However, the AMPT effects on brain metabolism and dopamine receptor occupancy have been studied in a series of previous studies,^16,47–50^ suggesting that AMPT mainly reduces dopamine neurotransmission in the striatum. This study did use other blood plasma as markers of biological processes mediating the behavioral response to AMPT treatment. This limits the interpretability of our findings, as interindividual metabolic as well as neural structural or functional differences may impact behavioral response to AMPT.

In summary, our findings suggest a relatively direct relationship between dopamine neurotransmission, and delusions and positive formal thought disorder in schizophrenia. They also suggest that dopamine neurotransmission is associated with impaired attribution of salience to reward-predicting stimuli that may underlie delusional symptoms in schizophrenia. More research is needed to examine the relationship between dopamine dysfunction, salience attribution, and psychotic symptoms in schizophrenia.

## Supplementary Material

sgad023_suppl_Supplementary_Material
