# Supplementary material for: Behavioral Response to Catecholamine Depletion in Individuals With Schizophrenia and Healthy Volunteers
Source: Schizophr Bull Open. 2023 Aug 24;4(1):sgad023. doi: 10.1093/schizbullopen/sgad023 (PMC11207692; doi:10.1093/schizbullopen/sgad023)
Supplement: sgad023_suppl_Supplementary_Material [file sgad023_suppl_Supplementary_Material.docx]

Suker et al. Behavioral response to catecholamine depletion in subjects with schizophrenia and healthy subjects.

**Supplementary Information**

**1. Supplementary Methods**

**Salience attribution test (SAT):**

The SAT is a paradigm that can be used to assess task-relevant and task-irrelevant motivational salience responses. We used the SAT as previously described by Roiser and colleagues^1^. Before testing, study participants were familiarized with the experiment in a tutorial. This tutorial comprised examples and two practice runs, which were not subject to monetary rewards. Participants were instructed that they should press a button as soon as possible after a square stimulus appears. The square was followed by a black screen for 2s after which one of four possible feedbacks was displayed: 1) “good”, when participants pressed the button before the square disappeared; 2) “Try to respond faster” if they responded after the square disappeared but before the feedback was shown, 3) “too early” when they pressed the button before the square was shown to them, and 4) “No key pressed” if participants did not press the button until the feedback was shown.

The mean reaction time in the first practice session was used as square presentation duration in the second practice session. Trials from the first practice session were rank-sorted according to response times, and the standard deviation for the fastest 50% of trials was calculated. This standard deviation was used to set the minimum and maximum square presentation durations for the second practice session (mean from first practice session ± 2xSD). The motivation for this was to avoid the skew due to very slow responses and, overall, to adjust task difficulty individually to each participant. For the subsequent test runs, mean, minimum and maximum stimulus (square) duration were set according to the mean, minimum, and maximum RT of the second practice run.

The test run consisted of two block each comprising 64 trials. At the beginning of each trial, a fixation cross was shown after 1000 ms. While the fixation cross remained on the screen, one of the four conditioned stimuli (CSs) was displayed at the top and bottom of the screen and remained there until the end of the trial. Each CS comprised two visual dimensions, color (blue and red) and shape (household objects, H; or animals, A). The proportion of trials with a reward varied over one of these dimensions (task-relevant), but not the other (task-irrelevant). There were four possible cues: blue H, blue A, red H, red A. However, only the cue color predicted the rewards: one color indicated a reward in 28 out of 32 of the trials (87.5%), the other color indicated a reward in 4 out of 32 of the trials (12.5%). Shape was irrelevant. We did not inform participants about these contingencies, and they had to learn which cue properties are relevant during the main runs of the experiment. After a variable period of time (between 0.5 and 1.5 s) a square was presented and participants had to respond as quickly as possible by pressing a button, before the disappearance of the square. Then, the square was followed by a black screen for 0.5 to 1.5 s. and the next trial began.

Subjects were informed about non-reinforced trials by a message displayed at the end of the trial: “Sorry, no money available.” In reinforced trials, participants received money according to their RT (from ¢5 to ¢100). When participants were better than their average calculated from the practice runs, they received the feedback: “Very good: ¢X”. The reward was calculated according to X=10+90 x (mean RT – trial RT)/(3xSDF), up to a maximum of 100¢; when they were slower than their average, the computer said: “Hit: ¢10”. When they failed to respond or responded after the target disappeared, they received the message: “Missed: ¢5”. When they responded prematurely, they received the feedback “Too early: ¢5”.

At the end of each block, participants had to indicate their estimate of the reinforcement probabilities (0-100) for each of the four different cues using a visual analogue scale (VAS).

Based on participants’ responses, we calculated two measures of motivational salience of the cues: 1) RT adaptive salience (implicit): RT of responses in high-probability-reinforcement trials subtracted from the RT for responses in low-probability-reinforcement trials. 2) VAS adaptive salience (explicit): probability rating for low-probability of reward trials subtracted from that for high-probability of reward trials. For each participant we averaged each of both measures from both blocks. We carried out our analyses with the averaged values.

**The n-back task:**

The n-back task is a continuous performance task that is commonly used as an assessment for working memory function. Participants started with a sensorimotor control task (0-back) during which they were required to respond positively by pressing the key 1 on the keyboard line when a pre-specified letter (x) was displayed and negatively by pressing key 2 when other letters were displayed. In the following, participants were required to indicate whether a letter presented on the screen (the ‘target’ stimulus) matched a previously presented letter (the ‘cue’ stimulus) by pressing either key 1 (positive response) or key 2 (negative response). The cue stimulus was defined as the letter presented either 1, 2, or 3 stimuli back for the 1, 2, and 3-back conditions, respectively.

Each condition consisted of a sequence of 30 letters. Letters were presented for 3500ms with a fixed interstimulus interval of 20ms. Prior to each letter sequence, an instruction screen indicated the task condition (0, 1-, 2- or 3-back). Each condition was presented three times in a row followed by the condition with the next higher cognitive load: three times 0-back, three times 1-back, three times 2-back, three times 3-back.

Statistical analysis was performed on the following dependent variables: number of hits, number of true negatives (correct rejections), number of false alarms (positive response to non-cue stimulus), number of false negatives (rejection of hits), omissions (trials without response), reaction time to hits and true negatives, reaction time to all correct responses, reaction time to all correct responses on a cue stimulus.

Analyses were carried out in 29 participants, 15 healthy controls and 14 participants with schizophrenia. One participant was excluded because only data from the first session was recorded.

**The Intra-dimensional/extra-dimensional set-shifting task (IDED):**

The IDED is a subtask of the Cambridge Neuropsychological Test Automated Battery (CANTAB) assessing adaptive learning ^2, 3^. Participants learn rules based on choices and subsequent feedback. Over nine different stages of the task, they have to learn new rules or notice and adapt to changes in previously learned rules.

The task was presented on a tablet. Participants had to choose between one of two options. They were instructed that only one of the two options is correct and that they would be provided with feedback (“correct” or “incorrect”) after pressing on one of the options. They were told that there is a rule they can learn in order to find the correct stimulus, but that this rule will change once it is apparent that they have understood the currently correct rule. The applicable rule changed eight times, i.e., the task consisted of nine different stages.

Successfully passing each stage required six consecutive correct choices. The test ended after completing all nine stages or if the subject failed to reach six consecutive correct choices over 50 attempts at any stage. Difficulty increased from stage 1 to stage 9. The first stage was a simple discrimination (SD) task, were participants had to choose from one of two stimuli of the same stimulus dimension (e.g., different lines). The second stage was a simple reversal (SR) task, where the previously correct element became incorrect, and vice versa. Thus, while stimuli remained the same in the first two stages, contingencies changed. Stage three, compound discrimination (C_D), introduced a distracting stimulus from a second category (shapes). Importantly, both categories, shapes and lines, were set apart and did not appear contiguous. In this stage, participants had to continue choosing the previously correct stimuli from the first category and had to ignore the new distracting stimuli from the second category. At stage four (CD), the same stimuli were presented, however, lines and shapes are superimposed to form more contiguous compound stimuli. Contingencies did not change in this stage. Stage five introduced a compound discrimination reversal (CDR): stimuli remained the same, but contingencies changed, such that the previously correct stimulus from the first dimension became the incorrect choice. The second stimulus category remained irrelevant. The sixth stage introduced an intra-dimensional shift (IDS). Here, new stimuli from the same two dimensions (lines and shapes) were introduced. Participants had to continue ignoring the second stimulus category and base their categorization solely on stimuli from the first dimension. At the seventh stage an intra-dimensional shift (IDR) occurred. Contingencies changed such that the element that was incorrect in the previous stage became the correct choice. Up until this stage only the first stimulus dimension determined which stimulus is the correct one. At stage eight an extra-dimensional shift (EDS) occurred. At this stage new exemplars from each stimulus dimension are presented. In addition, participants had to shift their attention from the previously relevant first stimulus dimension to the second stimulus dimension to find out the correct stimulus. Finally, stage nine introduced an extra-dimensional shift reversal (EDR). Participant had to shift to the previously incorrect stimulus from the second dimensions.

IDED parameters that we analyzed were the attrition rate, defined as the percentage of subjects who did not pass a stage, and the number of trials per stage, which is increasing by one with every error made ^4^.

**The handgrip-force task:**

Variations of this task have been implemented in fMRI and behavioral studies, consistently showing that it is a sensitive measure of behavioral effort-expenditure decision-making ^5-7^. The task was presented on a laptop computer. A grip force fiber optic response-device (dynamometer) was interfaced with a graphically displayed thermometer-like graphic on the computer screen. This “thermometer” presented the amount of grip force exerted by the participant on a scale of 0-10. The maximum force with which the dynamometer could be pressed was 400N.

First, maximum grip strength was individually calibrated. Participants were instructed to squeeze the grip device with their maximum strength 3 times with their dominant hand. They were told they could win up to 10 CHF if they pressed hard enough. Participants were shown a picture of a 10 CHF bill flowed by the “thermometer” and the challenge to press as hard as they can. This procedure was repeated three times. During each run, the pressure strength was recorded in 100 data points over approximately 3500ms. For each participant, the Mean value of the three runs (Mean1+Mean2+Mean3)/3=Mean) and the mean value of the three maximum strengths of the three runs (Max1+Max2+Max3)/3=Max) was calculated. Subsequently, the average of those values was calculated ((Mean+Max)/2). Subjects received the amount proportionately to the calculated force, where 400N would have corresponded to a payout of 10CHF.

After measuring individual grip strength, participants completed 45 test trials. Before each test trial, they had to decide whether they wanted to perform an "easy" task which was associated with the possibility of winning 1CHF or a "hard" task which was associated with the possibility of winning either CHF2, CHF5, or CHF10 with a probability of 13%, 50%, or 87%. On each trial, participants were informed about the amount to be won and the winning probability in the “hard” task (2CHF_13%, 5CHF_13%, 10CHF_13%, 2CHF_50%, 5CHF_50%, 10CHF_50%, 2CHF_87%, 5CHF_87%, 10CHF_87%). Each of these nine versions of the “hard” task was presented 5 times in random order, totaling in 45 trials.

The easy task involved squeezing the handgrip with 30% of the individual maximum grip force. The hard task required the subjects to squeeze the handgrip with at least 75% of their maximum strength for 3500ms, with 100 data points recorded. A red line in a thermometer-like graphic indicated to the participants the force requirements of the “easy” and “hard” tasks. If they started pressing too late or reduced their force too early the trial was scored as incorrect. At the end of each trial, participants were informed whether they were successful in pressing hard and long enough.

For the monetary reward actually paid out, subjects drew three tickets prior to the decision-making task. The first ticket determined the session (session 1 or 2), the other two tickets determined which two runs would be paid out. Subjects were informed about the payout rules but did not know which trials they picked to determine their payout.

The outcome of the decision-making task was the percentage of decisions for the hard task for each of the 9 “hard” conditions that were presented as an alternative to the same “easy” condition.

**2. Supplementary Results**

**Blinding**

After the first session when asked whether they received placebo or AMPT 10 out of 15 healthy controls and 9 out of 15 participants with schizophrenia guessed correctly. In healthy controls, all seven participants that received placebo correctly guessed the treatment, whereas only three out of eight correctly guessed that they were treated with AMPT. In participants with schizophrenia, four out of eight correctly guessed that they received placebo and five out of seven correctly guessed that they received AMPT.

After the second session when asked the same question 12 out of 15 healthy controls and 9 out of 15 participants with schizophrenia correctly guessed the treatment. In healthy controls, six out of eight participants correctly guessed that they received placebo and six out of seven correctly guessed that they received AMPT. In participants with schizophrenia, six out of seven participants correctly guessed that they received placebo and three out of eight correctly guessed they received AMPT.

**Supplementary Table 1. Effects of AMPT on SAPS Scores**

| **Scale / Effect** | **DFn** | **DFd** | **SSn** | **SSd** | **F** | **p** | **ges** |
| --- | --- | --- | --- | --- | --- | --- | --- |
| **Hallucinations** | |  |  |  |  |  |  |
| Intercept | 1 | 14 | 658.803 | 1019.739 | 9.045 | 0.009 | 0.383 |
| Treatment | 1 | 14 | 2.336 | 18.539 | 1.764 | 0.205 | 0.002 |
| Timepoint | 2 | 28 | 4.356 | 16.978 | 3.592 | 0.041 | 0.004 |
| Treatment:Timepoint | 2 | 28 | 1.156 | 6.844 | 2.364 | 0.113 | 0.001 |
| **Delusions** |  |  |  |  |  |  |  |
| Intercept | 1 | 14 | 11639.469 | 3803.739 | 42.840 | < 0.001 | 0.742 |
| Treatment | 1 | 14 | 5.136 | 120.072 | 0.599 | 0.452 | 0.001 |
| Timepoint | 2 | 28 | 89.306 | 69.111 | 18.091 | < 0.001 | 0.022 |
| Treatment:Timepoint | 2 | 28 | 18.406 | 50.511 | 5.101 | 0.013 | 0.005 |
| **Bizarre Behavior** | |  |  |  |  |  |  |
| Intercept | 1 | 14 | 74.711 | 88.289 | 11.847 | 0.004 | 0.421 |
| Treatment | 1 | 14 | 2.178 | 8.822 | 3.456 | 0.084 | 0.021 |
| Timepoint | 2 | 28 | 0.356 | 3.644 | 1.366 | 0.272 | 0.003 |
| Treatment:Timepoint | 2 | 28 | 0.089 | 1.911 | 0.651 | 0.529 | 0.001 |
| **Positive Formal Thought Disorder** | | | |  |  |  |  |
| Intercept | 1 | 14 | 3724.900 | 1552.600 | 33.588 | < 0.001 | 0.694 |
| Treatment | 1 | 14 | 9.344 | 30.822 | 4.244 | 0.058 | 0.006 |
| Timepoint | 2 | 28 | 12.867 | 29.133 | 6.183 | 0.006 | 0.008 |
| Treatment:Timepoint | 2 | 28 | 10.556 | 26.778 | 5.519 | 0.010 | 0.006 |

**Scale**, refers to SAPS scale (scores were calculated *without* the global rating score); **Effect**, refers to main effects and interactions; **DFn**, refers to nominator degrees of freedom; **DFd**, refers to denominator degrees of freedom; **SSn**, refers to nominator sum of squares; **SSd**, refers to denominator sum of squares; **F**, refers to F-statistic; **p**, refers to p-values, not corrected for sphericity; **ges**, refers to generalized effect size (n = 15 participants with schizophrenia). Sphericity corrections were carried out where indicated by Mauchly’s test. After sphericity corrections previously significant p-values remained significant (p < 0.05) with only one exception: the main effect Timepoint on the scale “Hallucinations” did not meet criteria for statistical significance (Greenhouse-Geisser corrected p-value = 0.063, Huynh-Feld corrected p-value = 0.06).

**Supplementary Table 2. Effects of AMPT on SANS Scores**

| **Scale / Effect** | **DFn** | **DFd** | **SSn** | **SSd** | **F** | **p** | **ges** |
| --- | --- | --- | --- | --- | --- | --- | --- |
| **Affective Flattening** | | | | | | | |
| Intercept | 1 | 14 | 2833.611 | 1625.556 | 24.404 | < 0.001 | 0.629 |
| Treatment | 1 | 14 | 0.278 | 17.556 | 0.222 | 0.645 | < 0.001 |
| Timepoint | 2 | 28 | 0.622 | 10.711 | 0.813 | 0.454 | < 0.001 |
| Treatment:Timepoint | 2 | 28 | 0.356 | 14.311 | 0.348 | 0.709 | < 0.001 |
| **Alogia** | | | | | | | |
| Intercept | 1 | 14 | 846.400 | 987.267 | 12.002 | 0.004 | 0.450 |
| Treatment | 1 | 14 | 0.044 | 30.289 | 0.021 | 0.888 | < 0.001 |
| Timepoint | 2 | 28 | 1.267 | 9.067 | 1.956 | 0.160 | 0.001 |
| Treatment:Timepoint | 2 | 28 | 0.289 | 7.378 | 0.548 | 0.584 | < 0.001 |
| **Apathy** | | | | | | | |
| Intercept | 1 | 14 | 2506.944 | 357.556 | 98.159 | < 0.001 | 0.872 |
| Treatment | 1 | 14 | 0.011 | 5.822 | 0.027 | 0.872 | < 0.001 |
| Timepoint | 2 | 28 | 0.289 | 2.711 | 1.492 | 0.242 | 0.001 |
| Treatment:Timepoint | 2 | 28 | 0.022 | 1.644 | 0.189 | 0.829 | < 0.001 |
| **Anhedonia - Asociality** | | | | | | | |
| Intercept | 1 | 14 | 6092.669 | 2324.206 | 36.700 | < 0.001 | 0.723 |
| Treatment | 1 | 14 | 0.803 | 7.406 | 1.518 | 0.238 | < 0.001 |
| Timepoint | 2 | 28 | 1.272 | 5.478 | 3.252 | 0.054 | 0.001 |
| Treatment:Timepoint | 2 | 28 | 0.672 | 2.744 | 3.429 | 0.047 | < 0.001 |
| **Attention** | | | | | | | |
| Intercept | 1 | 14 | 211.600 | 301.400 | 9.829 | 0.007 | 0.386 |
| Treatment | 1 | 14 | 0.400 | 11.267 | 0.497 | 0.492 | 0.001 |
| Timepoint | 2 | 28 | 3.267 | 13.733 | 3.330 | 0.050 | 0.010 |
| Treatment:Timepoint | 2 | 28 | 1.800 | 10.533 | 2.392 | 0.110 | 0.005 |

**Scale**, refers to SANS scale (scores were calculated *without* the global rating score); **Effect**, refers to main effects and interactions; **DFn**, refers to nominator degrees of freedom; **DFd**, refers to denominator degrees of freedom; **SSn**, refers to nominator sum of squares; **SSd**, refers to denominator sum of squares; **F**, refers to F-statistic; **p**, refers to p-value; **ges**, refers to generalized effect size (n = 15 participants with schizophrenia). Mauchly’s test indicated significant deviations from sphericity for the interaction effects Treatment*Timepoint on the scales “Anhedonia / Asociality” and “Attention”. For the interaction effect Treatment*Timepoint on “Anhedonia / Asociality” corrected p-values were as follows: Greenhouse-Geisser corrected p-value = 0.068, Huynh-Feld corrected p-value = 0.064. Sphericity-corrected p-values for the main effect Timepoint on “Attention” were: Greenhouse-Geisser corrected p-value = 0.069, Huynh-Feldt corrected p-value = 0.065.

**Supplementary Table 3. Effects of AMPT on MADRS Scores**

| **Effect** | **DFn** | | | **DFd** | **SSn** | **SSd** | **F** | **p** | **ges** |
| --- | --- | --- | --- | --- | --- | --- | --- | --- | --- |
| **Intercept** | | 1 | 27 | | 2831.123 | 2085.406 | 36.655 | < 0.001 | 0.384 |
| **Group** | | 1 | 27 | | 2186.801 | 2085.406 | 28.313 | < 0.001 | 0.481 |
| **Timepoint** | | 2 | 54 | | 7.378 | 45.059 | 4.421 | 0.017 | 0.002 |
| **Treatment** | | 1 | 27 | | 35.629 | 134.072 | 7.175 | 0.012 | 0.008 |
| **Group*Timepoint** | | 2 | 54 | | 0.459 | 45.059 | 0.275 | 0.761 | < 0.001 |
| **Group*Treatment** | | 1 | 27 | | 15.353 | 134.072 | 3.092 | 0.090 | 0.003 |
| **Timepoint*Treatment** | | 2 | 54 | | 1.270 | 75.649 | 0.453 | 0.638 | < 0.001 |
| **Group*Timepoint*Treatment** | | 2 | 54 | | 0.098 | 75.649 | 0.035 | 0.966 | < 0.001 |

**Effect**, refers to main effects and interactions; **DFn**, refers to nominator degrees of freedom; **DFd**, refers to denominator degrees of freedom; **SSn**, refers to nominator sum of squares; **SSd**, refers to denominator sum of squares; **F**, refers to F-statistic; **p**, refers to p-values, not corrected for sphericity; **ges**, refers to generalized effect size (n = 15 healthy controls and 14 participants with schizophrenia). After Mauchly’s test for sphericity, sphericity corrections were carried out for the interactions Timepoint*Treatment and Group*Timepoint*Treatment. These interactions remained non-significant (p > 0.5).

**Supplementary Table 4. Effects of AMPT on BAI Scores**

| **Effect** | **DFn** | **DFd** | **SSn** | **SSd** | **F** | **p** | **ges** |
| --- | --- | --- | --- | --- | --- | --- | --- |
| **Intercept** | 1 | 28 | 347.222 | 635.244 | 15.305 | 0.001 | 0.220 |
| **Group** | 1 | 28 | 231.200 | 635.244 | 10.191 | 0.003 | 0.188 |
| **Timepoint** | 2 | 56 | 29.811 | 164.422 | 5.077 | 0.009 | 0.024 |
| **Treatment** | 1 | 28 | 5.000 | 69.778 | 2.006 | 0.168 | 0.004 |
| **Group*Timepoint** | 2 | 56 | 9.100 | 164.422 | 1.550 | 0.221 | 0.007 |
| **Group*Treatment** | 1 | 28 | 8.889 | 69.778 | 3.567 | 0.069 | 0.007 |
| **Timepoint*Treatment** | 2 | 56 | 2.233 | 105.489 | 0.593 | 0.556 | 0.002 |
| **Group*Timepoint*Treatment** | 2 | 56 | 3.611 | 105.489 | 0.959 | 0.390 | 0.003 |

**Effect**, refers to main effects and interactions; **DFn**, refers to nominator degrees of freedom; **DFd**, refers to denominator degrees of freedom; **SSn**, refers to nominator sum of squares; **SSd**, refers to denominator sum of squares; **F**, refers to F-statistic; **p**, refers to p-values, not corrected for sphericity; **ges**, refers to generalized effect size (n = 15 healthy controls and 15 participants with schizophrenia). After Mauchly’s test for sphericity, sphericity corrections were carried out for the interactions Timepoint*Treatment and Group*Timepoint*Treatment. These interactions remained non-significant (p > 0.3).

**Supplementary Table 5. Relevant Bias in the Salience Attribution Task (SAT)**

|  | Dfn | DFd | SSn | SSd | F | p | ges |
| --- | --- | --- | --- | --- | --- | --- | --- |
| (Intercept) | 1 | 28 | 7248.473 | 12304.432 | 16.495 | < 0.001 | 0.250 |
| Group | 1 | 28 | 229.610 | 12304.432 | 0.523 | 0.476 | 0.011 |
| Treatment | 1 | 28 | 410.434 | 8126.071 | 1.414 | 0.244 | 0.019 |
| Group*Treatment | 1 | 28 | 1115.045 | 8126.071 | 3.842 | 0.060 | 0.051 |

**Effect**, refers to main effects and interactions; **DFn**, refers to nominator degrees of freedom; **DFd**, refers to denominator degrees of freedom; **SSn**, refers to nominator sum of squares; **SSd**, refers to denominator sum of squares; **F**, refers to F-statistic; **p**, refers to p-value; **ges**, refers to generalized effect size (n = 30, 15 participants of each group).

**Supplementary Table 6. Visual Analogue Scale for Adaptive Salience in the Salience Attribution Task (SAT)**

|  | Dfn | DFd | SSn | SSd | F | p | ges |
| --- | --- | --- | --- | --- | --- | --- | --- |
| (Intercept) | 1 | 28 | 64763.776 | 29287.292 | 61.917 | < 0.001 | 0.605 |
| Group | 1 | 28 | 910.651 | 29287.292 | 0.871 | 0.359 | 0.022 |
| Treatment | 1 | 28 | 39.609 | 10827.708 | 0.102 | 0.751 | 0.001 |
| Group*Treatment | 1 | 28 | 1203.776 | 10827.708 | 3.113 | 0.089 | 0.029 |

**Effect**, refers to main effects and interactions; **DFn**, refers to nominator degrees of freedom; **DFd**, refers to denominator degrees of freedom; **SSn**, refers to nominator sum of squares; **SSd**, refers to denominator sum of squares; **F**, refers to F-statistic; **p**, refers to p-value; **ges**, refers to generalized effect size (n = 30, 15 participants of each group).

**Supplementary Table 7. Irrelevant Bias in the Salience Attribution Task (SAT)**

|  | Dfn | DFd | SSn | SSd | F | p | ges |
| --- | --- | --- | --- | --- | --- | --- | --- |
| (Intercept) | 1 | 28 | 16958.137 | 8531.745 | 55.654 | < 0.001 | 0.545 |
| Group | 1 | 28 | 414.437 | 8531.745 | 1.360 | 0.253 | 0.029 |
| Treatment | 1 | 28 | 78.786 | 5043.235 | 0.437 | 0.514 | 0.006 |
| Group*Treatment | 1 | 28 | 168.173 | 5043.235 | 0.934 | 0.342 | 0.012 |

**Effect**, refers to main effects and interactions; **DFn**, refers to nominator degrees of freedom; **DFd**, refers to denominator degrees of freedom; **SSn**, refers to nominator sum of squares; **SSd**, refers to denominator sum of squares; **F**, refers to F-statistic; **p**, refers to p-value; **ges**, refers to generalized effect size (n = 30, 15 participants of each group).

**Supplementary Table 8. Visual Analogue Scale for Aberrant Salience in the Salience Attribution Task (SAT)**

| Effect | Dfn | DFd | SSn | SSd | F | p | ges |
| --- | --- | --- | --- | --- | --- | --- | --- |
| (Intercept) | 1 | 28 | 8431.276 | 3604.167 | 65.501 | < 0.001 | 0.572 |
| Group | 1 | 28 | 116.901 | 3604.167 | 0.908 | 0.349 | 0.019 |
| Treatment | 1 | 28 | 2.109 | 2540.833 | 0.023 | 0.880 | < 0.001 |
| Group*Treatment | 1 | 28 | 43.776 | 2540.833 | 0.482 | 0.493 | 0.007 |

**Effect**, refers to main effects and interactions; **DFn**, refers to nominator degrees of freedom; **DFd**, refers to denominator degrees of freedom; **SSn**, refers to nominator sum of squares; **SSd**, refers to denominator sum of squares; **F**, refers to F-statistic; **p**, refers to p-value; **ges**, refers to generalized effect size (n = 30, 15 participants of each group).

**Supplementary Table 9. Effects of Money Condition on Behavior in the Handgrip-Force Task**

| **Effect** | **DFn** | **DFd** | **SSn** | **SSd** | **F** | **p** | **ges** |
| --- | --- | --- | --- | --- | --- | --- | --- |
| (Intercept) | 1 | 28 | 59.168 | 3.716 | 445.824 | < 0.001 | 0.804 |
| Group | 1 | 28 | 1.618 | 3.716 | 12.193 | 0.002 | 0.112 |
| Treatment | 1 | 28 | 0.052 | 1.971 | 0.742 | 0.396 | 0.004 |
| Monetary reward | 2 | 56 | 1.801 | 0.802 | 62.889 | < 0.001 | 0.111 |
| Group*Treatment | 1 | 28 | 0.008 | 1.971 | 0.114 | 0.739 | 0.001 |
| Group*Monetary reward | 2 | 56 | 0.068 | 0.802 | 2.381 | 0.102 | 0.005 |
| Treatment*Monetary reward | 2 | 56 | 0.096 | 6.213 | 0.434 | 0.650 | 0.007 |
| Group*Treatment*Monetary reward | 2 | 56 | 0.015 | 6.213 | 0.069 | 0.934 | 0.001 |

**Effect**, refers to main effects and interactions; **DFn**, refers to nominator degrees of freedom; **DFd**, refers to denominator degrees of freedom; **SSn**, refers to nominator sum of squares; **SSd**, refers to denominator sum of squares; **F**, refers to F-statistic; **p**, refers to p-value; **ges**, refers to generalized effect size (n = 30, 15 participants per group). Mauchly’s test for Sphericity did not indicate a significant deviation from sphericity for the main effect “Monetary reward”.

**Supplementary Table 10. Effects of Probability Condition on Behavior in the Handgrip-Force Task**

| **Effect** | **DFn** | | | **DFd** | **SSn** | **SSd** | | | **F** | **p** | | **ges** | |  |
| --- | --- | --- | --- | --- | --- | --- | --- | --- | --- | --- | --- | --- | --- | --- |
| (Intercept) | | 1 | 28 | | 57.423 | | 3.573 | 450.005 | | | < 0.001 | | 0.806 | |
| Group | | 1 | 28 | | 1.922 | | 3.573 | 15.062 | | | 0.001 | | 0.139 | |
| Treatment | | 1 | 28 | | 0.118 | | 2.305 | 1.428 | | | 0.242 | | 0.008 | |
| Probability | | 2 | 56 | | 1.545 | | 1.058 | 40.861 | | | < 0.001 | | 0.100 | |
| Group*Treatment | | 1 | 28 | | 0.001 | | 2.305 | 0.007 | | | 0.932 | | < 0.001 | |
| Group*Probability | | 2 | 56 | | 0.019 | | 1.058 | 0.506 | | | 0.606 | | 0.001 | |
| Treatment*Probability | | 2 | 56 | | 0.185 | | 4.957 | 1.045 | | | 0.358 | | 0.013 | |
| Group*Treatment*Probability | | 2 | 56 | | 0.020 | | 4.957 | 0.112 | | | 0.894 | | 0.001 | |

**Effect**, refers to main effects and interactions; **DFn**, refers to nominator degrees of freedom; **DFd**, refers to denominator degrees of freedom; **SSn**, refers to nominator sum of squares; **SSd**, refers to denominator sum of squares; **F**, refers to F-statistic; **p**, refers to p-value; **ges**, refers to generalized effect size (n = 30, 15 participants per group). The main effect of “Monetary reward” remained significant after Sphericity correction (Greenhouse-Geisser corrected and Huynh-Feldt corrected p-values < 0.05). Mauchly’s test for Sphericity indicated a significant deviation from sphericity for the main effect “Monetary reward”. This effect remained significant after sphericity correction (p < 0.05).

**Supplementary Table 11. Number of Hits in the n-back Task**

| **Effect** | **DFn** | **DFd** | **SSn** | **SSd** | **F** | **p** | **ges** |
| --- | --- | --- | --- | --- | --- | --- | --- |
| (Intercept) | 1 | 27 | 130431.869 | 3698.200 | 952.263 | < 0.001 | 0.944 |
| Group | 1 | 27 | 1021.903 | 3698.200 | 7.461 | 0.011 | 0.132 |
| Treatment | 1 | 27 | 64.450 | 604.895 | 2.877 | 0.101 | 0.008 |
| Condition (0-back to 3-back) | 3 | 81 | 3701.217 | 1403.438 | 71.206 | < 0.001 | 0.323 |
| Group*Treatment | 1 | 27 | 6.622 | 604.895 | 0.296 | 0.591 | 0.001 |
| Group*Condition | 3 | 81 | 400.355 | 1403.438 | 7.702 | < 0.001 | 0.052 |
| Treatment*Condition | 3 | 81 | 87.772 | 583.676 | 4.060 | 0.010 | 0.011 |
| Group*Treatment*Condition | 3 | 81 | 21.255 | 583.676 | 0.983 | 0.405 | 0.003 |

**Effect**, refers to main effects and interactions; **DFn**, refers to nominator degrees of freedom; **DFd**, refers to denominator degrees of freedom; **SSn**, refers to nominator sum of squares; **SSd**, refers to denominator sum of squares; **F**, refers to F-statistic; **p**, refers to p-value; **ges**, refers to generalized effect size (n = 29, 15 healthy participants and 14 participants with schizophrenia). Results in the table are not corrected for sphericity violations. However, significant main effects and interaction effects (p < 0.05) remained significant after Huynh-Feldt and Greenhouse-Geisser corrections.

**Supplementary Table 12. Number of True Negatives in the n-back Task**

| **Effect** | | **DFn** | | **DFd** | | **SSn** | | **SSd** | | **F** | | **p** | | **ges** | |
| --- | --- | --- | --- | --- | --- | --- | --- | --- | --- | --- | --- | --- | --- | --- | --- |
| (Intercept) | 1 | | 27 | | 783934.232 | | 241.268 | | 87728.934 | | < 0.001 | | 0.998 | |  |
| Group | 1 | | 27 | | 31.611 | | 241.268 | | 3.538 | | 0.071 | | 0.020 | |  |
| Treatment | 1 | | 27 | | 2.168 | | 222.711 | | 0.263 | | 0.612 | | 0.001 | |  |
| Condition (0-back to 3-back) | 3 | | 81 | | 177.779 | | 465.239 | | 10.317 | | < 0.001 | | 0.103 | |  |
| Group*Treatment | 1 | | 27 | | 7.513 | | 222.711 | | 0.911 | | 0.348 | | 0.005 | |  |
| Group*Condition | 3 | | 81 | | 7.434 | | 465.239 | | 0.431 | | 0.731 | | 0.005 | |  |
| Treatment*Condition | 3 | | 81 | | 10.595 | | 560.491 | | 0.510 | | 0.676 | | 0.007 | |  |
| Group*Treatment*Condition | 3 | | 81 | | 15.595 | | 560.491 | | 0.751 | | 0.525 | | 0.010 | |  |

**Effect**, refers to main effects and interactions; **DFn**, refers to nominator degrees of freedom; **DFd**, refers to denominator degrees of freedom; **SSn**, refers to nominator sum of squares; **SSd**, refers to denominator sum of squares; **F**, refers to F-statistic; **p**, refers to p-value; **ges**, refers to generalized effect size (n = 29, 15 healthy participants and 14 participants with schizophrenia). Results in the table are not corrected for sphericity violations. However, significant main effects and interaction effects (p < 0.05) remained significant after Huynh-Feldt and Greenhouse-Geisser corrections.

**Supplementary Table 13. Number of False Alarms in the n-back Task**

| **Effect** | | **DFn** | | **DFd** | | **SSn** | | **SSd** | | **F** | | **p** | | **ges** | |
| --- | --- | --- | --- | --- | --- | --- | --- | --- | --- | --- | --- | --- | --- | --- | --- |
| (Intercept) | 1 | | 27 | | 499.679 | | 174.045 | | 77.516 | | < 0.001 | | 0.342 | |  |
| Group | 1 | | 27 | | 10.231 | | 174.045 | | 1.587 | | 0.219 | | 0.011 | |  |
| Treatment | 1 | | 27 | | 4.231 | | 118.045 | | 0.968 | | 0.334 | | 0.004 | |  |
| Condition (0-back to 3-back) | 3 | | 81 | | 168.828 | | 302.655 | | 15.061 | | < 0.001 | | 0.149 | |  |
| Group*Treatment | 1 | | 27 | | 0.438 | | 118.045 | | 0.100 | | 0.754 | | < 0.001 | |  |
| Group*Condition | 3 | | 81 | | 8.621 | | 302.655 | | 0.769 | | 0.515 | | 0.009 | |  |
| Treatment*Condition | 3 | | 81 | | 13.076 | | 346.131 | | 1.020 | | 0.388 | | 0.013 | |  |
| Group*Treatment*Condition | 3 | | 81 | | 1.421 | | 346.131 | | 0.111 | | 0.954 | | 0.001 | |  |

**Effect**, refers to main effects and interactions; **DFn**, refers to nominator degrees of freedom; **DFd**, refers to denominator degrees of freedom; **SSn**, refers to nominator sum of squares; **SSd**, refers to denominator sum of squares; **F**, refers to F-statistic; **p**, refers to p-value; **ges**, refers to generalized effect size (n = 29, 15 healthy participants and 14 participants with schizophrenia). Results in the table are not corrected for sphericity violations. However, significant main effects and interaction effects (p < 0.05) remained significant after Huynh-Feldt and Greenhouse-Geisser corrections.

**Supplementary Table 14. Number of False Negatives in the n-back Task**

| **Effect** | | **DFn** | **DFd** | **SSn** | | **SSd** | | **F** | | **p** | | **ges** | |
| --- | --- | --- | --- | --- | --- | --- | --- | --- | --- | --- | --- | --- | --- |
| (Intercept) | 1 | | 27 | 8587.153 | 3501.950 | | 66.207 | | < 0.001 | | 0.541 | |  |
| Group | 1 | | 27 | 961.981 | 3501.950 | | 7.417 | | 0.011 | | 0.132 | |  |
| Treatment | 1 | | 27 | 54.668 | 512.729 | | 2.879 | | 0.101 | | 0.007 | |  |
| Condition (0-back to 3-back) | 3 | | 81 | 3589.250 | 1402.026 | | 69.121 | | < 0.001 | | 0.330 | |  |
| Group*Treatment | 1 | | 27 | 1.702 | 512.729 | | 0.090 | | 0.767 | | < 0.001 | |  |
| Group*Condition | 3 | | 81 | 348.146 | 1402.026 | | 6.705 | | < 0.001 | | 0.048 | |  |
| Treatment*Condition | 3 | | 81 | 66.892 | 542.229 | | 3.331 | | 0.024 | | 0.009 | |  |
| Group*Treatment*Condition | 3 | | 81 | 13.168 | 542.229 | | 0.656 | | 0.582 | | 0.002 | |  |

**Effect**, refers to main effects and interactions; **DFn**, refers to nominator degrees of freedom; **DFd**, refers to denominator degrees of freedom; **SSn**, refers to nominator sum of squares; **SSd**, refers to denominator sum of squares; **F**, refers to F-statistic; **p**, refers to p-value; **ges**, refers to generalized effect size (n = 29, 15 healthy participants and 14 participants with schizophrenia). Results in the table are not corrected for sphericity violations. However, main effects and interaction effects (p < 0.05) remained significant after Huynh-Feldt and Greenhouse-Geisser corrections.

**Supplementary Table 15. IDED Attrition Collapsed over Both Sessions**

|  |  | AMPT | Placebo |
| --- | --- | --- | --- |
| Stage 8 | Healthy Controls | 2 | 3 |
| Stage 8 | Participants with Schizophrenia | 4 | 2 |
| Stage 9 | Healthy Controls | 1 | 1 |
| Stage 9 | Participants with Schizophrenia | 0 | 0 |

**Supplementary Table 16. IDED Attrition for Each Session**

|  |  |  | AMPT | Placebo |
| --- | --- | --- | --- | --- |
| Session 1 | Stage 8 | Healthy Controls | 2 | 0 |
| Session 1 | Stage 8 | Participants with Schizophrenia | 3 | 1 |
| Session 1 | Stage 9 | Healthy Controls | 1 | 0 |
| Session 1 | Stage 9 | Participants with Schizophrenia | 0 | 0 |
| Session 2 | Stage 8 | Healthy Controls | 0 | 3 |
| Session 2 | Stage 8 | Participants with Schizophrenia | 1 | 1 |
| Session 2 | Stage 9 | Healthy Controls | 0 | 1 |
| Session 2 | Stage 9 | Participants with Schizophrenia | 0 | 0 |

**Supplementary Table 17. Analyses of the IDED (Stage 1-7)**

| Effect | Dfn | Dfd | SSn | SSd | F | p | ges |
| --- | --- | --- | --- | --- | --- | --- | --- |
| (Intercept) | 1 | 28 | 22381.800 | 449.543 | 1394.062 | < 0.001 | 0.897 |
| Group | 1 | 28 | 49.371 | 449.543 | 3.075 | 0.090 | 0.019 |
| Stage | 6 | 168 | 184.133 | 1205.257 | 4.278 | < 0.001 | 0.067 |
| Treatment | 1 | 28 | 0.467 | 76.819 | 0.170 | 0.683 | < 0.001 |
| Group*Stage | 6 | 168 | 52.895 | 1205.257 | 1.229 | 0.294 | 0.021 |
| Group*Treatment | 1 | 28 | < 0.001 | 76.819 | < 0.001 | > 0.999 | < 0.001 |
| Stage*Treatment | 6 | 168 | 10.467 | 715.314 | 0.410 | 0.872 | 0.004 |
| Group*Stage*Treatment | 6 | 168 | 21.933 | 715.314 | 0.859 | 0.527 | 0.009 |

**Effect**, refers to main effects and interactions; **DFn**, refers to nominator degrees of freedom; **DFd**, refers to denominator degrees of freedom; **SSn**, refers to nominator sum of squares; **SSd**, refers to denominator sum of squares; **F**, refers to F-statistic; **p**, refers to p-value; **ges**, refers to generalized effect size (n = 30, 15 participants of each group). **Stage**, refers to the main effect of stage with only stages 1-7 included in the analysis. Results in the table are not corrected for **sphericity** violations. After sphericity correction the main effect of stage remained significant (p = 0.031, Greenhouse-Geisser correction; p = 0.029, Hynh-Feldt correction). After sphericity corrections the following interactions effects remained non-significant (p > 0.05): Group*Stage, Stage*Treatment, Group*Stage*Treatment.

**Supplementary Table 18. Correlations between MWT and SANS / SAPS**

| **Correlation test** | **Treatment** | **rho** | **p** | **n** |
| --- | --- | --- | --- | --- |
| MWT x AFlattening | 0 | -0.069 | 0.816 | 14 |
| MWT x Alogia | 0 | 0.109 | 0.711 | 14 |
| MWT x Apathy | 0 | -0.029 | 0.921 | 14 |
| MWT x Anhedonia | 0 | -0.051 | 0.864 | 14 |
| MWT x Attention | 0 | 0.101 | 0.730 | 14 |
| MWT x Hallucinations | 0 | -0.258 | 0.374 | 14 |
| MWT x Delusions | 0 | 0.243 | 0.403 | 14 |
| MWT x BizBehavior | 0 | 0.128 | 0.664 | 14 |
| MWT x PFTDisorder | 0 | 0.049 | 0.867 | 14 |
| MWT x AFlattening | 1 | -0.123 | 0.675 | 14 |
| MWT x Alogia | 1 | 0.153 | 0.601 | 14 |
| MWT x Apathy | 1 | -0.009 | 0.976 | 14 |
| MWT x Anhedonia | 1 | -0.009 | 0.976 | 14 |
| MWT x Attention | 1 | 0.088 | 0.764 | 14 |
| MWT x Hallucinations | 1 | -0.221 | 0.448 | 14 |
| MWT x Delusions | 1 | 0.055 | 0.852 | 14 |
| MWT x BizBehavior | 1 | -0.135 | 0.646 | 14 |
| MWT x PFTDisorder | 1 | 0.060 | 0.837 | 14 |

**MWT**, refers to the sum of correct responses in the verbal test Mehrfachwahl-Wortschatztest, version B; **SANS scores at Timepoint 0**, as follows: **AFlattening**, refers to affective flattening, **Alogia**, **Apathy**, **Anhedonia**, and **Attention** referring to the corresponding scales; **SAPS scores at Timepoint 0**, as follows: **Hallucinations** and **Delusions** refer to the corresponding scales, **BizBehavior** refers to Bizzare behavior, **PFTDisorder**, refers to positive formal thought disorder; **Treatment** refers to pharmacological treatment with 0 denoting placebo and 1 denoting AMPT; **rho**, refers to Spearman’s rho estimation, **p-value**, refers to the corresponding two-tailed p-value, uncorrected for multiple comparisons, n refers to the number of valid cases.

**Supplementary Table 19. Correlations between DSST and SANS / SAPS**

| **Correlation test** | **Treatment** | **rho** | **p** | **n** |
| --- | --- | --- | --- | --- |
| DSST x AFlattening | 0 | -0.048 | 0.870 | 14 |
| DSST x Alogia | 0 | -0.059 | 0.842 | 14 |
| DSST x Apathy | 0 | -0.088 | 0.764 | 14 |
| DSST x Anhedonia | 0 | -0.049 | 0.867 | 14 |
| DSST x Attention | 0 | -0.036 | 0.904 | 14 |
| DSST x Hallucinations | 0 | -0.038 | 0.897 | 14 |
| DSST x Delusions | 0 | 0.156 | 0.595 | 14 |
| DSST x BizBehavior | 0 | -0.205 | 0.481 | 14 |
| DSST x PFTDisorder | 0 | 0.070 | 0.812 | 14 |
| DSST x AFlattening | 1 | -0.028 | 0.925 | 14 |
| DSST x Alogia | 1 | -0.039 | 0.894 | 14 |
| DSST x Apathy | 1 | -0.072 | 0.808 | 14 |
| DSST x Anhedonia | 1 | -0.064 | 0.829 | 14 |
| DSST x Attention | 1 | 0.064 | 0.827 | 14 |
| DSST x Hallucinations | 1 | -0.011 | 0.970 | 14 |
| DSST x Delusions | 1 | 0.144 | 0.624 | 14 |
| DSST x BizBehavior | 1 | 0.075 | 0.798 | 14 |
| DSST x PFTDisorder | 1 | 0.105 | 0.722 | 14 |

**DSST**, refers to the number of correct responses in the Digit Symbol Substitution subtest of Wechsler Adult Intelligence Scale; **SANS**, as follows: **AFlattening**, refers to affective flattening, **Alogia**, **Apathy**, **Anhedonia**, and **Attention** referring to the corresponding scales; **SAPS**, as follows: **Hallucinations** and **Delusions** refer to the corresponding scales, **BizBehavior** refers to Bizzare behavior, **PFTDisorder**, refers to positive formal thought disorder; **Treatment** refers to pharmacological treatment with 0 denoting placebo and 1 denoting AMPT; **rho**, refers to Spearman’s rho estimation, **p-value**, refers to the corresponding two-tailed p-value, uncorrected for multiple comparisons, n refers to the number of valid cases.

**Supplementary Table 20. Correlations between n-back task performance and SANS / SAPS**

| **Correlation test** | **Condition** | **rho** | **p** | **n** |
| --- | --- | --- | --- | --- |
| n-back Hits x AFlattening | 0 | 0.053 | 0.857 | 14 |
| n-back Hits x Alogia | 0 | 0.435 | 0.120 | 14 |
| n-back Hits x Apathy | 0 | -0.115 | 0.694 | 14 |
| n-back Hits x Anhedonia | 0 | 0.094 | 0.748 | 14 |
| n-back Hits x Attention | 0 | -0.185 | 0.526 | 14 |
| n-back Hits x Hallucinations | 0 | -0.060 | 0.839 | 14 |
| n-back Hits x Delusions | 0 | 0.478 | 0.084 | 14 |
| n-back Hits x BizBehavior | 0 | 0.306 | 0.287 | 14 |
| n-back Hits x PFTDisorder | 0 | 0.101 | 0.731 | 14 |
| n-back Hits x AFlattening | 1 | -0.051 | 0.863 | 14 |
| n-back Hits x Alogia | 1 | 0.012 | 0.969 | 14 |
| n-back Hits x Apathy | 1 | -0.304 | 0.291 | 14 |
| n-back Hits x Anhedonia | 1 | 0.367 | 0.197 | 14 |
| n-back Hits x Attention | 1 | 0.043 | 0.883 | 14 |
| n-back Hits x Hallucinations | 1 | -0.119 | 0.684 | 14 |
| n-back Hits x Delusions | 1 | -0.186 | 0.524 | 14 |
| n-back Hits x BizBehavior | 1 | -0.186 | 0.524 | 14 |
| n-back Hits x PFTDisorder | 1 | -0.290 | 0.315 | 14 |
| n-back Hits x AFlattening | 2 | -0.225 | 0.439 | 14 |
| n-back Hits x Alogia | 2 | -0.061 | 0.836 | 14 |
| n-back Hits x Apathy | 2 | -0.006 | 0.985 | 14 |
| n-back Hits x Anhedonia | 2 | 0.262 | 0.366 | 14 |
| n-back Hits x Attention | 2 | -0.054 | 0.854 | 14 |
| n-back Hits x Hallucinations | 2 | 0.312 | 0.277 | 14 |
| n-back Hits x Delusions | 2 | 0.110 | 0.707 | 14 |
| n-back Hits x BizBehavior | 2 | -0.205 | 0.482 | 14 |
| n-back Hits x PFTDisorder | 2 | -0.048 | 0.871 | 14 |
| n-back Hits x AFlattening | 3 | -0.199 | 0.496 | 14 |
| n-back Hits x Alogia | 3 | -0.108 | 0.713 | 14 |
| n-back Hits x Apathy | 3 | -0.246 | 0.397 | 14 |
| n-back Hits x Anhedonia | 3 | 0.401 | 0.155 | 14 |
| n-back Hits x Attention | 3 | -0.054 | 0.854 | 14 |
| n-back Hits x Hallucinations | 3 | 0.448 | 0.108 | 14 |
| n-back Hits x Delusions | 3 | 0.306 | 0.287 | 14 |
| n-back Hits x BizBehavior | 3 | -0.248 | 0.392 | 14 |
| n-back Hits x PFTDisorder | 3 | -0.071 | 0.809 | 14 |

**n-back Hits**, refers to the number of hits in the n-back task under **Condition** 0-3, referring to 0-back, 1-back, 2-back, and 3-back, correspondingly; n-back-task performance refers to **placebo treatment**; **SANS**, as follows: **AFlattening**, refers to affective flattening, **Alogia**, **Apathy**, **Anhedonia**, and **Attention** referring to the corresponding scales; **SAPS**, as follows: **Hallucinations** and **Delusions** refer to the corresponding scales, **BizBehavior** refers to Bizzare behavior, **PFTDisorder**, refers to positive formal thought disorder; all SANS and SAPS scores refer to **Timepoint 0 under placebo treatment**; **rho**, refers to Spearman’s rho estimation, **p-value**, refers to the corresponding two-tailed p-value, uncorrected for multiple comparisons, n refers to the number of valid cases.

**Supplementary Figure 1. SAPS: Only Participants With Schizophrenia That Were Administered Placebo First**

***Blue dots*** represent rating scores of individual participants; ***red lines*** connect group averages per timepoint; ***time points*** as follows: 0 = baseline, 1 = 27 hours after the begin of AMPT/placebo administration, 2 = 51 hours after the begin of AMPT/placebo administration; ***sample size***, n = 7 participants with schizophrenia.

**Supplementary Figure 2.** **SAPS: Only Participants With Schizophrenia That Were Administered AMPT First**

***Blue dots*** represent rating scores of individual participants; ***red lines*** connect group averages per timepoint; ***time points*** as follows: 0 = baseline, 1 = 27 hours after the begin of AMPT/placebo administration, 2 = 51 hours after the begin of AMPT/placebo administration; ***sample size***, n = 8 participants with schizophrenia.

**Supplementary Figure 3. SAPS in Healthy Controls**

***Blue dots*** represent rating scores of individual participants; ***red lines*** connect group averages per timepoint; ***time points*** as follows: 0 = baseline, 1 = 27 hours after the begin of AMPT/placebo administration, 2 = 51 hours after the begin of AMPT/placebo administration; ***sample size***, n = 15 healthy controls.

**Supplementary Figure 4. SANS In Participants With Schizophrenia**

***Blue dots*** represent rating scores of individual participants; ***red lines*** connect group averages per timepoint; ***time points*** as follows: 0 = baseline, 1 = 27 hours after the begin of AMPT/placebo administration, 2 = 51 hours after the begin of AMPT/placebo administration; ***sample size***, n = 15 participants with schizophrenia.

**Supplementary Figure 5. SANS: Only Participants With Schizophrenia That Were Administered Placebo First**

***Blue dots*** represent rating scores of individual participants; ***red lines*** connect group averages per timepoint; ***time points*** as follows: 0 = baseline, 1 = 27 hours after the begin of AMPT/placebo administration, 2 = 51 hours after the begin of AMPT/placebo administration; ***sample size***, n = 7 participants with schizophrenia.

**Supplementary Figure 6. SANS: Only Participants With Schizophrenia That Were Administered AMPT First**

***Blue dots*** represent rating scores of individual participants; ***red lines*** connect group averages per timepoint; ***time points*** as follows: 0 = baseline, 1 = 27 hours after the begin of AMPT/placebo administration, 2 = 51 hours after the begin of AMPT/placebo administration; ***sample size***, n = 8 participants with schizophrenia.

**Supplementary Figure 7. SANS: Healthy Participants**

***Blue dots*** represent rating scores of individual participants; ***red lines*** connect group averages per timepoint; ***time points*** as follows: 0 = baseline, 1 = 27 hours after the begin of AMPT/placebo administration, 2 = 51 hours after the begin of AMPT/placebo administration; ***sample size***, n = 15 healthy participants.

**Supplementary Figure 8. Effects of AMPT on MADRS and BAI**

***Blue dots*** represent rating scores of individual participants; ***red lines*** connect group averages per timepoint; ***time points*** as follows: 0 = baseline, 1 = 27 hours after the begin of AMPT/placebo administration, 2 = 51 hours after the begin of AMPT/placebo administration; ***sample size***, for MADRS analysis n = 29, with 15 healthy controls and 14 participants with schizophrenia; for BAI analysis n = 30 with 15 participants per group.

**Supplementary Figure 9**

Three hours after the end of AMPT treatment:

Spearman’s rho = -0.7612319; p-value = 0.0009786 (two-tailed)

Pearson’s r = -0.6010606; p-value = 0.01779 (two-tailed)

**Supplementary Figure 10**


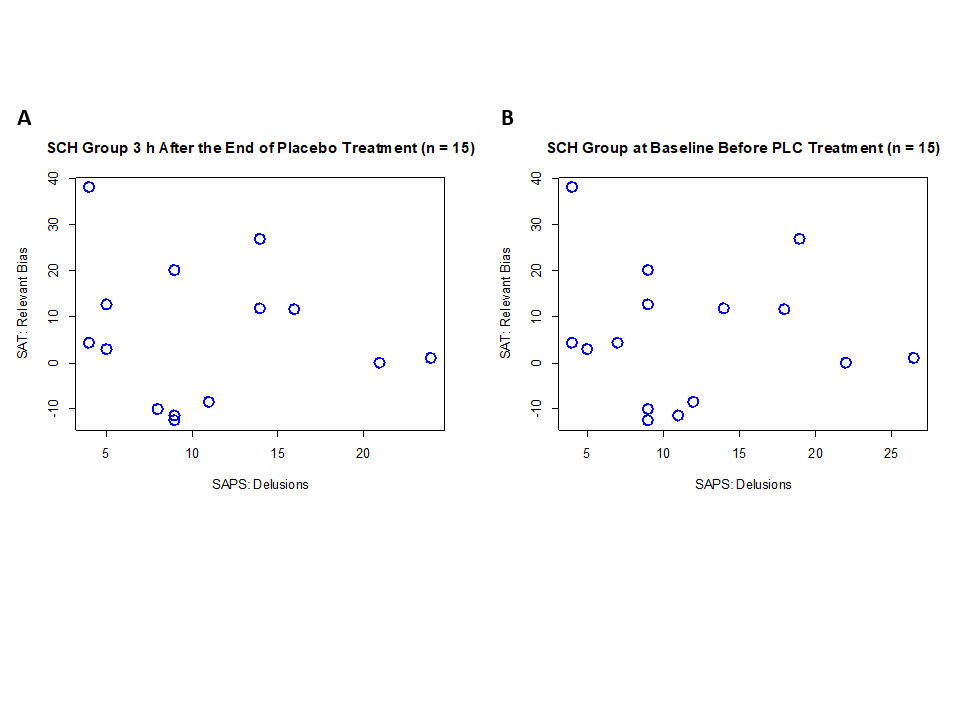


***A.*** Three hours after the end of placebo administration:

Spearman’s rho = -0.1693762; p-value = 0.5462 (two-tailed)

Pearson’s r = -0.1163321; p-value = 0.6797 (two-tailed)

***B.*** At baseline before placebo administration:

Spearman’s rho = -0.1496918; p-value = 0.5944 (two-tailed)

Pearson’s r = -0.07008026; p-value = 0.804 (two-tailed)

**Supplementary Figure 11. Force Task: Effect of Money Condition**

***Blue circles*** represent individual participants; ***red lines*** connect group averages per task condition (here, the amount to win); ***sample size***, n = 30 participants (15 healthy controls and 15 participants with schizophrenia).

**Supplementary Figure 12. Force Task: Effects of Money Condition and Treatment**

***Blue circles*** represent individual participants; ***red lines*** connect group averages per task condition (here, the amount to win); ***sample size***, n = 30 participants (15 healthy controls and 15 participants with schizophrenia).

**Supplementary Figure 13. Force Task: Effects of Probability Condition**

***Blue circles*** represent individual participants; ***red lines*** connect group averages per task condition (here, the probability of winning); ***sample size***, n = 30 participants (15 healthy controls and 15 participants with schizophrenia).

**Supplementary Figure 14. Force Task: Effects of Probability Condition and Treatment**

***Blue circles*** represent individual participants; ***red lines*** connect group averages per task condition (here, the probability of winning); ***sample size***, n = 30 participants (15 healthy controls and 15 participants with schizophrenia).

**Supplementary Figure 15. Performance in the n-back Task. Number of Correct Rejections (True Negatives)**

***Blue circles*** represent individual participants; ***red lines*** connect group averages across conditions; ***sample size***, n = 29 participants (15 healthy controls and 14 participants with schizophrenia).

**Supplementary Figure 16. Performance in the n-back Task. Number of False Alarms (False Positives).**

***Blue circles*** represent individual participants; ***red lines*** connect group averages across conditions; ***sample size***, n = 29 participants (15 healthy controls and 14 participants with schizophrenia).

**Supplementary Figure 17. Performance in the n-back Task. Number of False Negatives (Wrong Rejections)**

***Blue circles*** represent individual participants; ***red lines*** connect group averages across conditions; ***sample size***, n = 29 participants (15 healthy controls and 14 participants with schizophrenia).

**Supplementary Figure 18. Performance in the IDED**

***Blue circles*** represent individual participants; ***red lines***, connect group averages under placebo and AMPT treatment (missing red lines for stage 9 indicate that at least one participant did not enter this condition); ***Stages*** as follows: ***SD***, simple discrimination; ***SR***, simple reversal; ***C_D***, compound discrimination; ***CD***, compound discrimination of contiguous stimuli; ***CDR***, compound discrimination reversal; ***IDS***, intra-dimensional shift; ***IDR***, intra-dimensional shift reversal; ***EDS***, extra-dimensional shift; ***EDR***, extra-dimensional shift reversal. ***sample size***, n = 30 participants (15 healthy controls and 15 participants with schizophrenia). All subjects completed the first seven stages. Five healthy controls did not successfully complete stage 8 (2 receiving AMPT and 3 receiving placebo); six participants with schizophrenia did not successfully complete stage 8 (4 receiving AMPT and 3 receiving placebo). Two healthy controls did not complete stage 9 (one receiving AMPT and the other placebo).

**References**

**1.** Roiser JP, Stephan KE, den Ouden HE, Barnes TR, Friston KJ, Joyce EM. Do patients with schizophrenia exhibit aberrant salience? *Psychol Med* Feb 2009;39(2):199-209.

**2.** Sahakian BJ, Owen AM. Computerized assessment in neuropsychiatry using CANTAB: discussion paper. *J R Soc Med* Jul 1992;85(7):399-402.

**3.** Downes JJ, Roberts AC, Sahakian BJ, Evenden JL, Morris RG, Robbins TW. Impaired extra-dimensional shift performance in medicated and unmedicated Parkinson's disease: evidence for a specific attentional dysfunction. *Neuropsychologia* 1989;27(11-12):1329-1343.

**4.** Jazbec S, Pantelis C, Robbins T, Weickert T, Weinberger DR, Goldberg TE. Intra-dimensional/extra-dimensional set-shifting performance in schizophrenia: Impact of distractors. *Schizophrenia research* Jan 2007;89(1-3):339-349.

**5.** Pessiglione M, Schmidt L, Draganski B, Kalisch R, Lau H, Dolan RJ, Frith CD. How the brain translates money into force: a neuroimaging study of subliminal motivation. *Science* May 11 2007;316(5826):904-906.

**6.** Kurniawan IT, Seymour B, Talmi D, Yoshida W, Chater N, Dolan RJ. Choosing to make an effort: the role of striatum in signaling physical effort of a chosen action. *J Neurophysiol* Jul 2010;104(1):313-321.

**7.** Reddy LF, Horan WP, Barch DM, et al. Effort-Based Decision-Making Paradigms for Clinical Trials in Schizophrenia: Part 1-Psychometric Characteristics of 5 Paradigms. *Schizophrenia bulletin* Sep 2015;41(5):1045-1054.
